# Expanding Synthetic Lethality in DNA Damage Response-Defective Cancers Through Stress Phenotype-Guided Kinase Targeting

**DOI:** 10.3390/ijms27188301

**Published:** 2026-09-17

**Authors:** Mirco Masi, Giulia Varignani, Andrea Cavalli, Stefania Girotto

**Affiliations:** 1 Computational and Chemical Biology, Italian Institute of Technology (IIT), 16163 Genoa, Italy; giulia.varignani@iit.it (G.V.); andrea.cavalli@iit.it (A.C.); 2 Department of Pharmacy and Biotechnology, University of Bologna, 40126 Bologna, Italy; 3 Centre Européen de Calcul Atomique et Moléculaire (CECAM), Ecole Polytechnique Fédérale de Lausanne, 1015 Lausanne, Switzerland; 4 Structural Biophysics Facility, Italian Institute of Technology (IIT), 16163 Genoa, Italy

**Keywords:** synthetic lethality, DNA damage response (DDR), homologous recombination deficiency (HRD), stress-adaptive kinases, replication stress, chromosomal instability, PARP inhibitors, patient stratification, proteolysis-targeting chimera (PROTAC), precision oncology

## Abstract

Synthetic lethality has reshaped oncology, particularly in tumours with defects in DNA damage response (DDR) pathways, yet therapeutic strategies centred on canonical DDR targets, including poly(ADP-ribose) polymerase (PARP), ataxia telangiectasia and Rad3-related protein (ATR), checkpoint kinase 1 (CHK1) and Wee1 G2 checkpoint kinase (WEE1), remain constrained by resistance, toxicity and biological heterogeneity of DDR alterations. Emerging evidence indicates that DDR deficiency extends beyond impaired DNA repair to generate interconnected stress phenotypes involving replication fork instability, chromosomal instability, transcriptional and cell-cycle dysregulation, oxidative/proteotoxic stress and metabolic imbalance. These states may increase tumour-cell reliance on kinases that are not canonical DNA repair enzymes or proximal DDR sensors, here referred to as non-canonical DDR-associated kinases. In this review, we examine the mechanistic rationale and translational potential of targeting kinases that regulate mitosis, transcriptional adaptation, checkpoint signalling, stress responses and metabolic homeostasis in DDR-defective tumours. We distinguish kinase dependencies supported by direct DDR-context-specific vulnerability or PARP inhibitor sensitisation from those whose relevance remains primarily mechanistic or hypothesis-generating and propose a shift from genotype-based patient selection toward functional stress phenotyping that integrates DNA repair capacity with replication stress, chromosomal instability, transcriptional conflict and the metabolic state. Finally, we evaluate pharmacological strategies, including rational combinations, allosteric modulation, polypharmacology and targeted protein degradation, and highlight the need for biomarker-guided clinical translation.

## 1. Introduction

Synthetic lethality arises when the simultaneous dysfunction of two genes results in cell death, while the impairment of either gene alone does not compromise cell viability [1]. This paradigm has progressively reshaped the therapeutic landscape in oncology, particularly in the context of tumours harbouring defects in DNA damage response (DDR) pathways [2]. By orchestrating the detection and repair of DNA lesions, DDR mechanisms safeguard genome integrity and chromosomal stability to preserve cellular homeostasis [3]. Upon genetic or functional impairment of one DNA repair pathway, cancer cells become over-reliant on compensatory mechanisms for survival, thus creating selective vulnerabilities that can be therapeutically exploited through synthetic lethality [3]. The most paradigmatic example of this concept is represented by the clinical success of poly(ADP-ribose) polymerase (PARP) inhibitors (PARPi) in different tumour types harbouring germline or somatic mutations of breast cancer susceptibility gene 1 (*BRCA1*) and *BRCA2*. While PARP enzymes are required for the efficient repair of DNA single-strand breaks (SSBs) through base- (BER) and nucleotide excision repair (NER), BRCA1 and BRCA2—together with the DNA recombinase radiation sensitive protein 51 (RAD51)—are pivotal mediators of homologous recombination (HR)-directed repair of DNA double-strand breaks (DSBs) [1]. In BRCA-deficient or BRCAness-like contexts, PARP inhibition leads to the accumulation of unrepaired DNA lesions, replication fork collapse, chromosomal instability (CIN) and, ultimately, cell death through synthetic lethality [4,5,6,7]. The success of PARPi established a broader DDR-centric therapeutic framework and stimulated the investigation of additional synthetic lethal targets within DNA repair signalling networks. In particular, master DDR sensor kinases ataxia-telangiectasia mutated (ATM), ataxia-telangiectasia and Rad3-related protein (ATR) and DNA-dependent protein kinase (DNA-PK), as well as other DDR kinases like checkpoint kinase 1 (CHK1) and Wee1 G2 checkpoint kinase (WEE1), have emerged as key therapeutic candidates [2]. Within this context, HR deficiency (HRD), whose testing is crucial for identifying patients who will benefit from targeted DNA-damaging cancer therapies, represents the most clinically validated model of DDR-related synthetic lethality [7]. However, broader forms of DDR impairment, including *ATM* loss, replication checkpoint dysfunction or DNA-PK alterations, generate overlapping stress phenotypes with HRD [8].

Despite the relevance of targeting PARP and canonical DDR sensor kinases, several limitations have progressively emerged. First, resistance to PARPi and other DDR-targeting agents frequently develops through different mechanisms, including HR restoration, replication fork stabilisation, and adaptive rewiring of compensatory signalling pathways [9]. Second, direct inhibition of core DDR enzymes may result in dose-limiting toxicities, given their fundamental role in genome maintenance also in normal cells [10]. Third, HRD itself represents a heterogeneous condition, with *BRCA1/2* mutations as the most representative ones, but also encompassing alterations in genes like partner and localiser of BRCA2 (*PALB2*) and *RAD51* paralogs (*RAD51B*, *RAD51C*, *RAD51D*, X-ray repair cross-complementing protein 2 (*XRCC2*) and *XRCC3*), epigenetic silencing events and functional deficiencies detectable through genomic scar signatures, thus complicating patient stratification and therapeutic predictability [11,12]. These considerations collectively suggest that synthetic lethal vulnerabilities in DDR-defective cancer cells may extend beyond canonical DDR repair proteins and proximal DDR sensors to encompass adaptive signalling networks activated in response to the multifaceted consequences of HR/DDR impairment [13].

Several previous reviews have addressed DDR-based synthetic lethality strategies focusing primarily on canonical DNA repair proteins and proximal DDR targets, including PARP, ATM, ATR, DNA-PK, CHK1 and WEE1, on HRD testing and PARPi resistance, or on kinase inhibitors as broader pharmacological classes [13,14,15,16]. Conversely, the present review takes a different perspective. Rather than considering DDR alterations only as gene-centric biomarkers or direct drug targets, we use them as entry points to define functional stress phenotypes that may reshape tumour-cell dependencies. We here define “non-canonical DDR-associated kinases” as kinases that are neither canonical DNA repair enzymes nor proximal DDR sensors/transducers, and whose relevance in DDR-defective tumours is proposed to arise primarily from their role in tolerating DDR-associated cellular stress rather than from direct DNA lesion recognition or repair. This operational definition excludes canonical DDR-directed targets (e.g., PARP, ATM, ATR, DNA-PK, CHK1 and WEE1, here discussed only as contextual comparators) from the main scope of the review. Conversely, it includes kinases regulating mitotic tolerance, transcriptional and cell-cycle adaptation, checkpoint rewiring, stress-response signalling or metabolic/redox homeostasis when experimental evidence, therapeutic sensitisation data or mechanistic rationale links them to DDR-associated stress states [17,18,19,20]. Borderline cases, particularly cyclin-dependent kinases 12 and 13 (CDK12/13), that are not DNA lesion sensors or repair enzymes, but directly influence DDR competence through transcriptional control of repair and cell-cycle genes are treated explicitly as interface kinases.

In this view, DDR impairment should not be considered exclusively as a defect in DNA repair capacity, but rather as a cellular state that promotes distinct yet interconnected stress phenotypes, including replication-associated genome instability, transcriptional and cell-cycle imbalance, proteotoxic/oxidative stress, and metabolic dysregulation. These stress phenotypes may impose selective evolutionary pressure on tumour cells, thereby increasing their reliance on signalling networks that coordinate mitotic progression, transcriptional adaptation, stress-response signalling or metabolic homeostasis. However, evidence supporting individual kinase dependencies remains highly heterogeneous. In some cases, selective vulnerabilities have been experimentally validated in well-defined DDR-defective backgrounds; in others, evidence is limited to sensitisation effects in combination with PARPi or DNA-damaging agents, or to mechanistic associations with DDR-related stress adaptation pathways. By integrating DDR alterations with stress phenotypes and the current evidence supporting specific kinase dependencies, we propose a stress-oriented and evidence-aware framework that moves beyond gene-centric models of synthetic lethality and more comprehensively captures tumour vulnerabilities in DDR-defective malignancies. Rather than providing an exhaustive catalogue of the human kinome, the aim of this review is to highlight representative kinase axes through which DDR-defective tumours may acquire stress-adaptive vulnerabilities and to discuss how these vulnerabilities could inform biomarker-guided therapeutic strategies.

## 2. DDR Impairment as a Generator of Stress-Adaptive Kinase Dependencies

**Figure 1:**
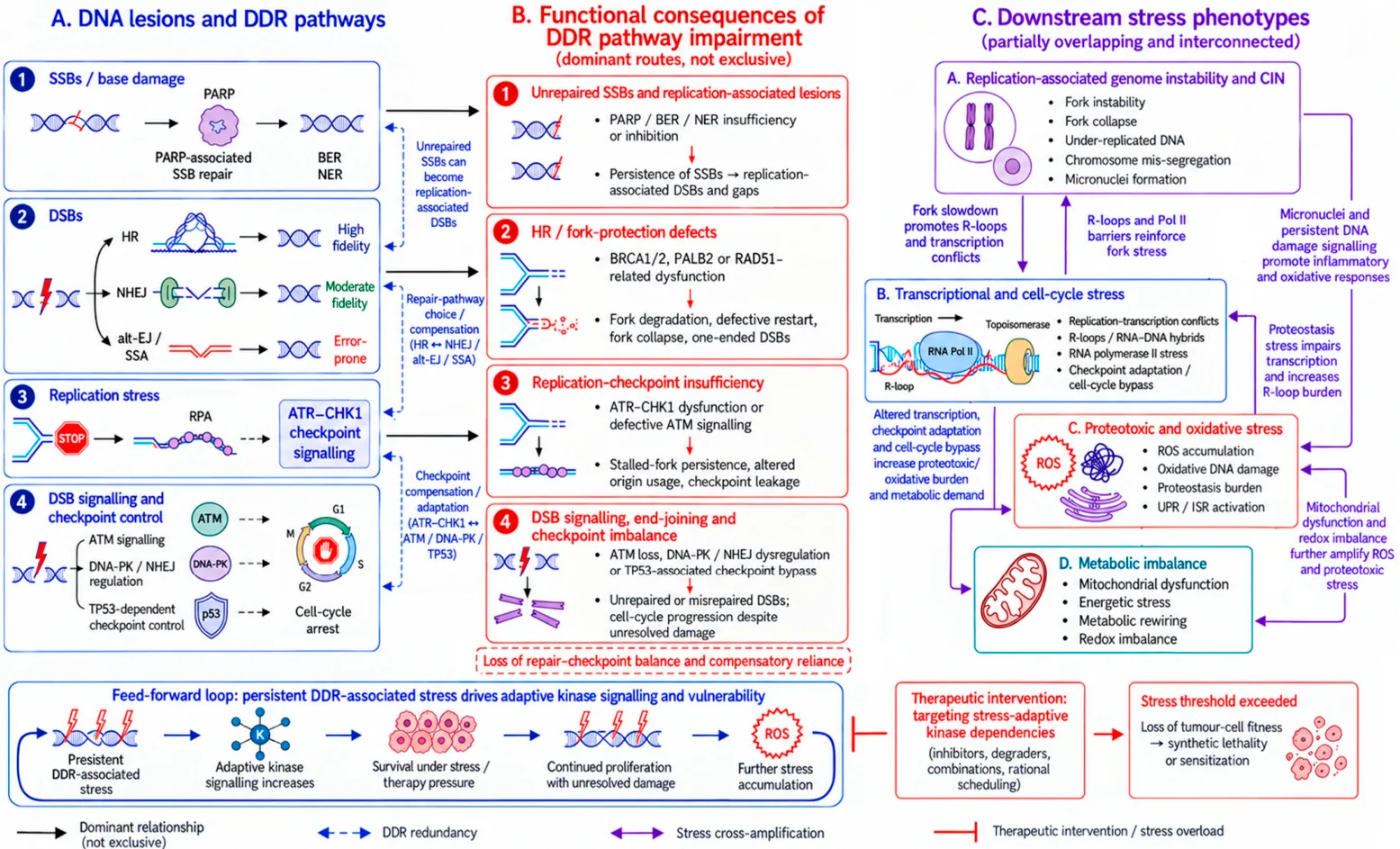
**From DDR pathway defects to interconnected stress phenotypes**. DDR pathways preserve genome integrity by coordinating DNA lesion recognition, repair and cell-cycle control. SSBs and base damage are mainly processed through PARP-associated SSB repair and BER/NER, whereas DSBs can be repaired through HR (high fidelity), NHEJ (moderate fidelity), alt-EJ or SSA (error-prone). In parallel, ATR–CHK1 signalling coordinates replication stress responses, while ATM, DNA-PK and TP53-dependent checkpoint control regulate DSB signalling, end-joining balance and cell-cycle arrest. Defects in these pathways are not equivalent, but can generate dominant functional consequences, including unrepaired SSBs and replication-associated lesions, HR/fork-protection defects, replication-checkpoint insufficiency, defective repair–checkpoint balance, unrepaired or misrepaired DSBs and cell-cycle progression despite unresolved damage. These consequences converge on partially overlapping downstream stress phenotypes, including replication-associated genome instability and CIN, transcriptional and cell-cycle stress, proteotoxic/oxidative stress and metabolic imbalance. In particular, fork slowdown/collapse and under-replicated DNA may promote replication–transcription conflicts, while R-loops and RNA polymerase II barriers can reinforce fork stress; micronuclei and persistent DNA damage signalling may promote inflammatory and oxidative responses; altered transcription, checkpoint adaptation and proteostasis stress may reinforce proteotoxic/oxidative burden, R-loop accumulation and metabolic demand; and mitochondrial dysfunction and redox imbalance may further amplify reactive oxygen species (ROS), proteotoxic stress and energetic imbalance. Persistent DDR-associated stress may therefore establish a feed-forward loop in which adaptive kinase signalling supports survival under unresolved genomic stress or therapy pressure, allowing continued proliferation and further stress accumulation. Targeting the kinases regulating these stress-adaptive dependencies may push DDR-defective tumour cells beyond a tolerable stress threshold, promoting synthetic lethality or therapeutic sensitisation. Hence, this framework provides the mechanistic basis for considering DDR-defective tumours as stress-adapted cellular states rather than as simple DNA repair-deficient entities (solid black arrows indicate dominant relationships that are not intended to be exclusive; dashed blue arrows indicate DDR pathway redundancy or compensation; purple arrows indicate evidence-supported stress cross-amplifying interactions among stress phenotypes, rather than a universal chronological cascade; red arrows indicate therapeutic intervention or stress overload).

DNA integrity is continuously challenged by endogenous and exogenous insults that generate different types of lesions requiring coordinated recognition, signalling and repair. To safeguard genome integrity, cells have evolved an integrated network of DDR pathways that detect damage, promote its repair and coordinate cell-cycle progression accordingly [21]. DDR pathways—including PARP-associated SSB repair as well as DSB pathways like high-fidelity HR and error-prone single-strand annealing (SSA), non-homologous end joining (NHEJ) and alternative end-joining (alt-EJ)—and replication checkpoint signalling preserve genome integrity by coupling lesion repair with cell-cycle control [3,21]. HR is a high-fidelity DNA repair mechanism restricted to S and G2 phases that exploits the sister chromatid as a template to ensure the accurate resolution of DSBs, replication-associated lesions and stalled replication forks. BRCA1, BRCA2 and RAD51 act as central mediators of this pathway, orchestrating DNA end resection, RAD51 filament formation and strand invasion, thereby ensuring genome stability [11]. The coordinated engagement of these pathways reflects a dynamic balance between repair efficiency and accuracy, influenced by cell-cycle stage, chromatin context and regulatory signalling [3,21]. However, defects in HR, ATM signalling, replication checkpoint control or end-joining pathways disrupt this balance, leading to persistent DNA lesions, replication-associated damage, progressive genomic instability and chromosomal aberrations [3,11]. Importantly, the consequences of DDR impairment extend beyond defective DNA repair itself. HRD and broader DDR impairment forms generate interconnected stress phenotypes that collectively reshape cancer cell dependencies and promote reliance on compensatory signalling networks [17,18,19,20]. Figure 1 provides a schematic representation of the major DDR pathways affected in DDR-defective tumours, illustrating how defects in repair and checkpoint pathways disrupt the balance between DNA repair and checkpoint control, thereby giving rise to interconnected cellular stress states.

### 2.1. Replication-Associated Genome Instability and Chromosomal Instability

Upon impairment of HR-associated or fork-protection factors, replication checkpoint dysfunction (ATR/CHK1 axis) or defective ATM signalling, replication-associated stress emerges as one of the earliest and most prominent consequences. However, the mechanisms leading to fork instability are not identical across DDR-defective contexts. In this review, replication fork instability is used as an umbrella term encompassing increased susceptibility of stalled forks to nucleolytic degradation or resection, defective fork restart, fork collapse, and altered checkpoint-mediated control of replication dynamics. Importantly, the fork-protection functions of BRCA1/2 and RAD51-related factors can be partially separable from their canonical role in HR-directed DSB repair [22]. For instance, BRCA2 protects nascent DNA at stalled forks from DSB repair protein meiotic recombination (MRE11)-dependent degradation independently of homology-directed repair, while separation-of-function models such as the RAD51 T131P mutation suggest that RAD51-dependent genome maintenance defects can occur despite preserved HR capacity [23,24]. This separation is further supported by evidence that BRCA1–BRCA1-associated RING domain protein 1 (BARD1) promotes replication fork protection through mechanisms that are not equivalent to canonical BRCA1–PALB2-mediated HR, with cancer-associated BRCA1–BARD1 variants displaying defective protection of nascent DNA strands while retaining HR proficiency [25]. Therefore, fork instability in DDR-defective tumours should not be equated with HR failure alone, but may reflect defective fork protection, excessive nucleolytic processing, impaired fork restart or checkpoint insufficiency.

DDR-impaired cells can display stalled replication forks that are susceptible to nucleolytic degradation, leading to fork collapse, formation of one-ended DSBs and increased replication-associated DNA damage [26,27]. This instability is frequently accompanied by compensatory increased origin firing, which further exacerbates replication stress and promotes the depletion of nucleotide pools [28]. Moreover, in replication checkpoint-defective settings, loss of ATR–CHK1-mediated restraint can promote unscheduled or dormant origin firing, as shown by primary studies demonstrating that ATR/CHK1 inhibition induces aberrant origin activation through CDC7-dependent origin firing and RIF1–PP1-associated mechanisms [29,30]. Thus, altered origin usage should be interpreted as a context-dependent consequence of impaired replication-checkpoint control rather than as a universal feature of all HRD or BRCA-deficient contexts. A persistent, unresolved replication stress can propagate into mitosis, where under-replicated DNA, unresolved repair intermediates and structural chromosome abnormalities promote chromosome mis-segregation [27]. Indeed, HRD and broader DDR-impaired cells display an increased frequency of anaphase bridges, lagging chromosomes and micronuclei formation [31]. These mitotic abnormalities are not just a consequence of defects in DNA repair, but can further amplify genomic instability, DNA damage signalling and inflammatory responses [32]. In this regard, HRD/DDR-defective cells may become more reliant on mitotic checkpoint regulators and CIN-tolerance mechanisms, suggesting that kinases controlling mitotic progression, spindle assembly and chromosome segregation may represent therapeutically relevant vulnerabilities [33]. Altogether, replication fork instability, checkpoint-dependent alterations in origin usage and chromosomal mis-segregation define a state of chronic replication-associated genome instability, supporting the rationale for targeting mitotic and CIN-tolerance kinases as potential vulnerabilities in DDR-defective tumours.

### 2.2. Transcriptional and Cell-Cycle Stress

Transcriptional programs and cell-cycle regulation are other mechanisms heavily affected by HRD and broader DDR impairment [34]. Cellular backgrounds characterised by genomic instability usually display exacerbated replication–transcription conflicts, particularly when replication forks encounter highly transcribed regions or persistent RNA–DNA hybrids. As a result of these collisions, R-loops and additional DNA lesions are generated, further amplifying genomic stress [35]. These mechanisms are supported by primary studies showing that topoisomerase I-deficient cells accumulate R-loops, stalled replication forks, H2A histone family member X (H2AX) phosphorylation and chromosome breaks, which can be suppressed by preventing RNA–DNA hybrid formation [36]. Similarly, engineered human-cell systems demonstrated that head-on transcription–replication conflicts increase R-loop levels and activate orientation-specific DNA damage responses [37], while R-loop processing by transcription-coupled nucleotide excision repair factors can generate DSBs [38]. More recently, unresolved transcription–replication conflicts were shown to contribute to PARPi-mediated lethality in HR-deficient cells [39], supporting the relevance of this mechanism in DDR-defective contexts. Thus, replication–transcription conflicts provide a concrete source of R-loop-associated fork stress and DNA damage in DDR-defective contexts.

Chronic or aberrant DDR signalling may promote checkpoint adaptation, allowing cells to bypass cell-cycle arrest despite persistent DNA damage, thus favouring survival at the expense of genomic fidelity [40]. DDR-defective tumours may also display an altered expression of genes involved in cell-cycle progression, transcriptional control and stress responses [41]. In this context, transcriptional cyclin-dependent kinases (CDKs) and other regulators of RNA polymerase II activity may become particularly important for maintaining transcriptional homeostasis and stress-adaptive gene expression. The inhibition of transcriptional and cell-cycle regulatory kinases may therefore exacerbate replication–transcription conflicts, impair the expression of stress-response genes or disrupt residual checkpoint adaptation. However, the consequences of targeting these kinases may vary depending on the specific DDR lesion, tumour lineage and functional stress state. In some settings, kinase inhibition may produce selective vulnerability, enhance sensitivity to PARPi and DNA-damaging agents, or remain primarily mechanistic, as presented in the following sections. This distinction is particularly relevant for kinases lying at the interface between transcriptional regulation, cell-cycle progression and genome maintenance.

### 2.3. Proteotoxic, Oxidative and Metabolic Stress

In addition to replication-associated perturbations, HRD and broader DDR impairments are also associated with substantial redox and metabolic alterations [42]. DNA damage accumulation and unresolved replication stress have been reported to increase reactive oxygen species (ROS) production [42]. This, in turn, can further damage both nuclear and mitochondrial DNA, thereby establishing a self-reinforcing cycle of genomic instability and oxidative imbalance [43]. In parallel, persistent genotoxic stress and altered protein synthesis demands may activate proteostasis pathways, including unfolded protein response (UPR) and integrated stress response (ISR) signalling [44], thereby creating additional reliance on stress-adaptation kinases [45]. Mitochondrial dysfunction and altered Krebs cycle activity have also been described in HRD/DDR-impaired cells, strengthening the link between energy metabolism and DNA repair capacity [46,47]. Literature data indicate that cancer cells exposed to persistent or aberrant DDR activation can modulate metabolic fluxes, amino acid consumption and nucleotide biosynthesis pathways, which ultimately influence redox homeostasis [47,48]. Considering that oxidative stress can both result from and contribute to genomic instability [43], DDR-defective cells may become increasingly dependent on signalling kinases involved in metabolic adaptation, ROS buffering, mitochondrial function and proteotoxic stress control [42]. In this regard, several metabolic sensors have been implicated in the coordination of energy stress responses with cell-cycle progression and DNA repair processes [49]. Similarly, stress-response pathways such as ISR, mitogen-activated protein kinase (MAPK)-associated checkpoint signalling and survival kinase networks may contribute to cellular adaptation under chronic genotoxic stress [50]. Therefore, proteotoxic, oxidative and metabolic imbalance induced by DDR impairment may represent additional layers of vulnerability extending beyond direct DNA repair mechanisms.

### 2.4. Implications for Non-Canonical DDR-Associated Kinase Dependencies

Taken together, HRD and broader DDR impairment should not be viewed only as defective DSB repair states, but as multifaceted cellular conditions characterised by replication-associated genome instability, CIN, transcriptional and cell-cycle dysregulation, oxidative stress, proteotoxic burden and metabolic imbalance [13]. These stress phenotypes are interconnected rather than independent, since replication stress can promote chromosomal mis-segregation and micronuclei formation, oxidative stress can further increase DNA damage, and transcriptional conflicts can reinforce replication-associated instability [13]. Mitotic kinases may support division under conditions of CIN; transcriptional and cell-cycle kinases may sustain stress-adaptive gene expression and checkpoint adaptation; stress-response kinases may buffer proteotoxic and oxidative damage; and metabolic kinases may preserve energetic and redox homeostasis. However, these dependencies are not equivalent in terms of experimental support or therapeutic maturity. For some kinases, selective vulnerability in DDR-defective backgrounds or robust sensitisation to PARPi and DNA-damaging agents has been demonstrated. For others, the current rationale is based mainly on mechanistic links to DDR-associated stress phenotypes. Nevertheless, this stress-oriented perspective provides a rationale for why DDR-defective tumour cells may become dependent on non-canonical DDR-associated kinases.

## 3. Non-Canonical DDR-Associated Kinases as Stress-Adaptive Vulnerabilities in DDR-Defective Tumours

**Figure 2:**
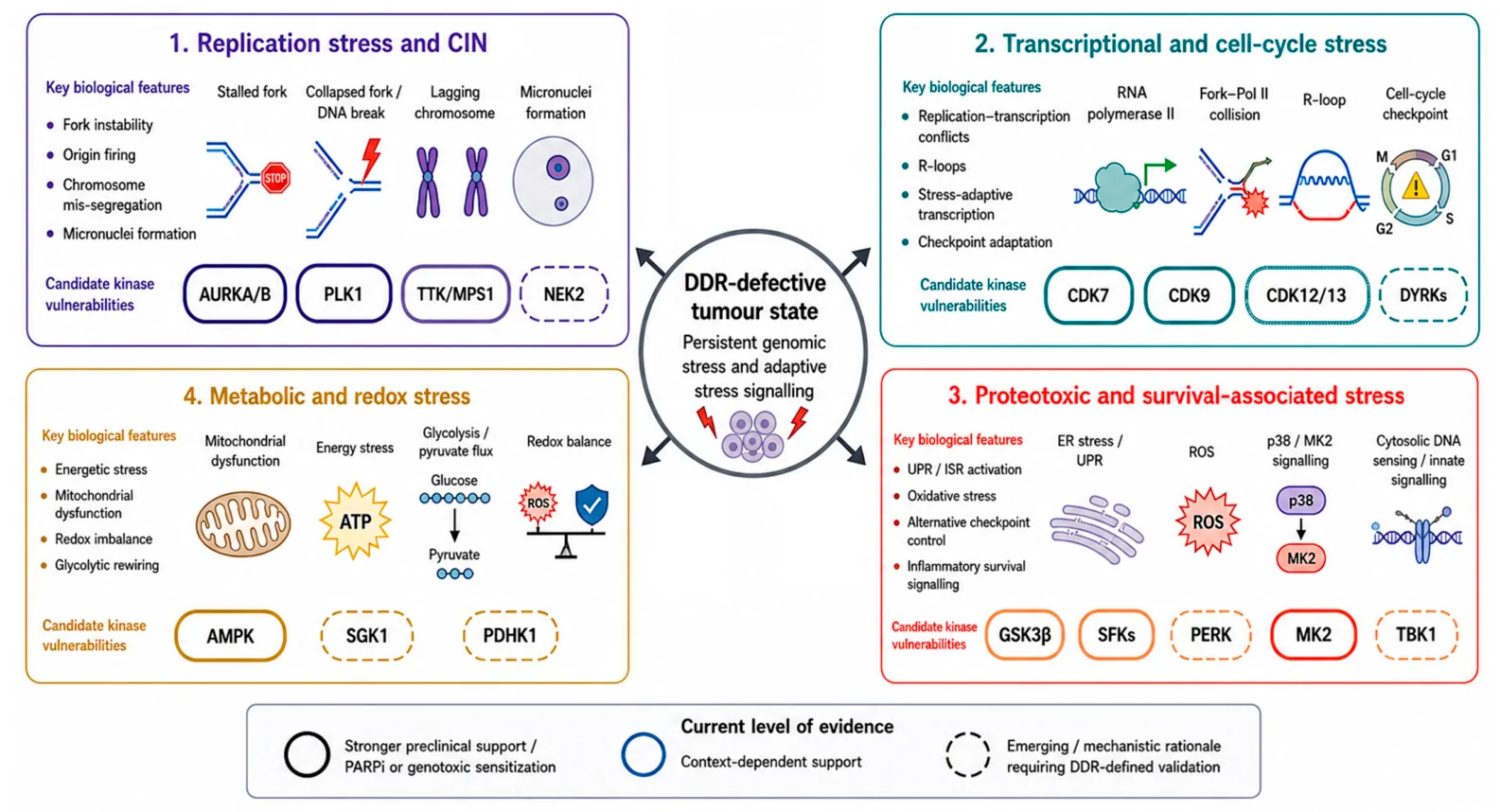
**Stress-phenotype map of non-canonical DDR-associated kinase vulnerabilities.** DDR-defective tumours display heterogeneous yet interconnected stress phenotypes that may create context-dependent reliance on distinct kinase networks. Replication stress and CIN, characterised by fork instability, aberrant origin firing, chromosome mis-segregation and micronuclei formation, provide a rationale for targeting mitotic and CIN-tolerance kinases such as Aurora kinase A/B (AURKA/B), polo-like kinase 1 (PLK1), monopolar spindle kinase 1 (TTK/MPS1) and NIMA-related kinase 2 (NEK2). Transcriptional and cell-cycle stress, driven by replication–transcription conflicts, R-loop accumulation, stress-adaptive transcription and checkpoint adaptation, may increase reliance on cyclin-dependent kinase 7 (CDK7), CDK9, CDK12/13 and dual-specificity tyrosine-phosphorylation-regulated kinases (DYRKs), with CDK12/13 representing a borderline DDR-transcription interface rather than purely non-DDR targets. Proteotoxic, checkpoint and survival stress may involve UPR/ISR activation, oxidative stress, alternative checkpoint control and inflammatory survival signalling, implicating glycogen synthase kinase 3 beta (GSK3β), Src family kinases (SFKs), protein kinase R (PKR)-like endoplasmic reticulum kinase (PERK), MAPK-activated protein kinase 2 (MK2) and TANK-binding kinase 1 (TBK1) as adaptive nodes with variable degrees of support. Metabolic and redox stress, including energetic stress, mitochondrial dysfunction, redox imbalance and glycolytic rewiring, may involve AMP-activated protein kinase (AMPK), serum- and glucocorticoid-regulated kinase 1 (SGK1) and pyruvate dehydrogenase kinase (PDHK1). Visual coding indicates the current level of evidence, distinguishing kinase vulnerabilities with stronger preclinical or PARPi/genotoxic sensitisation evidence from context-dependent or emerging candidates requiring DDR-defined validation. The map emphasises that these kinases should not be interpreted as uniformly validated synthetic lethal targets, but as stress-adaptive vulnerabilities whose relevance depends on tumour context, functional stress state and biomarker support.

As discussed above, HRD and broader DDR impairment establish a complex cellular state extending beyond DSB repair, where cancer cells are forced to cope with persistent replication stress, CIN, transcriptional imbalance, oxidative stress and metabolic perturbations [13]. In this context, non-canonical DDR-associated kinases may contribute to tumour cell adaptation by sustaining mitotic progression, transcriptional programs, stress-response signalling or metabolic homeostasis [17,18,19,20]. However, as previously stated in the Introduction section, the available evidence supporting these kinase dependencies is heterogeneous. Accordingly, the kinases discussed below should not be interpreted as uniformly validated synthetic lethal targets, but as stress-adaptive vulnerabilities whose relevance depends on the DDR lesion, tumour lineage and functional stress state. The kinases included in this section were selected according to three criteria: (i) they are not canonical DNA repair enzymes or proximal DDR sensors/transducers; (ii) their biological functions map to at least one DDR-associated stress phenotype discussed in Section 2; and (iii) published experimental evidence, therapeutic sensitisation data or mechanistic rationale links them to DDR-defective or genotoxic-stress contexts. Therefore, the list is representative rather than exhaustive and is intended to illustrate stress-phenotype-specific kinase axes rather than to provide a comprehensive catalogue of the kinome. An overview of DDR impairment-associated stress phenotypes and the corresponding non-canonical DDR-associated kinase vulnerabilities is summarised in Figure 2.

### 3.1. Mitotic and Chromosome Instability Tolerance Kinases

HR-deficient tumours, as well as cancers harbouring *ATM* loss or replication checkpoint dysfunction, frequently exhibit increased CIN levels [12,27,51], which arises as a consequence of replication fork collapse, incomplete DNA repair and aberrant segregation of structurally altered chromosomes [52]. Persistent DNA lesions that are not correctly repaired during the S phase can propagate into mitosis, resulting in the formation of different CIN hallmarks like anaphase bridges, lagging chromosomes and micronuclei formation [31]. Multiple lines of evidence indicate that these mitotic abnormalities represent a strong selective pressure for the activation of checkpoint and spindle assembly mechanisms that safeguard chromosome segregation fidelity [53]. In this context, kinases orchestrating mitotic progression, spindle assembly and checkpoint surveillance may become particularly important for the survival of tumour cells experiencing DDR impairment-associated CIN, creating potential vulnerabilities beyond canonical DDR components (Figure 2).

#### 3.1.1. Aurora Kinase A (AURKA) and AURKB

AURKA and AURKB are serine/threonine kinases that play a pivotal role in centrosome maturation, spindle assembly and chromosome alignment [54]. While AURKA is mostly involved in regulating mitotic entry, centrosome maturation/separation and bipolar spindle assembly, AURKB controls chromosome condensation, biorientation and segregation as well as cytokinesis as part of the chromosomal passenger complex [54]. Due to their frequent overexpression in human tumours and their role in carcinogenesis, Aurora kinases emerged as interesting drug targets [54] also in the context of synthetic lethality [55,56]. Within the DDR context, the relationship between AURKA and BRCA2/HR should be interpreted cautiously, as the available evidence does not support a simple unidirectional model. Previous studies reported an inverse relationship between AURKA and BRCA2, where AURKA overexpression is associated with reduced BRCA2 expression, while AURKA silencing can restore BRCA2 expression and increase BRCA2/RAD51 DNA damage repair foci following irradiation [57,58], suggesting a link between AURKA activity and BRCA2-dependent repair capacity. However, subsequent work showed that inhibition of AURKA, either by gene silencing or alisertib exposure, can mimic a BRCAness-like state, by increasing NHEJ activity, activating DNA-PK, and reducing BRCA1/2 expression, together with increased γH2AX levels, induction of DSBs and enhanced PARPi sensitivity [59]. Therefore, AURKA should be viewed as a context-dependent regulator of DNA repair pathway balance and genome-stability tolerance rather than as a kinase with a simple inverse relationship to BRCA2. In HR/DDR-deficient or CIN-high cells, such perturbation of repair-pathway balance may converge with the accumulation of structural chromosomal aberrations, reducing the tolerance to additional mitotic stress. In this setting, Aurora kinases’ inhibition may exacerbate chromosome segregation defects and promote mitotic catastrophe. This rationale may extend beyond classical HRD to broader DDR-impaired contexts, including Fanconi Anaemia pathway alterations [60], where a synthetic lethal interaction between AURKA and Fanconi anaemia complementation group A (FANCA) has been suggested [61]. Several preclinical studies also support an enhanced vulnerability of genomically unstable tumours to Aurora kinase inhibition [62,63,64,65,66]. Overall, the available evidence supports Aurora kinases as mitotic stress-buffering vulnerabilities in CIN-high DDR-impaired tumours, particularly in settings of HRD, Fanconi Anaemia pathway dysfunction or *ATM* loss [63]. However, most data support an enhanced vulnerability or therapeutic sensitisation rather than a defined synthetic lethal relationship with DDR deficiency.

#### 3.1.2. Polo-like Kinase 1 (PLK1)

As a master regulator of mitotic progression, PLK1 controls centrosome maturation, spindle formation and cytokinesis [67]. Highly proliferative and genomically unstable tumours frequently overexpress PLK1, which in turn induces CIN and suppresses the DDR pathway [68,69], while contrasting evidence indicates that PLK1 overexpression may also exert tumour-suppressive properties [70], suggesting that its impact on cancer development and progression may be tissue-dependent. Nevertheless, PLK1 inhibition has been reported to induce pronounced mitotic arrest and apoptosis in different cancer contexts [71], also within paradigms of classical synthetic lethality and synthetic dosage lethality, a genetic interaction where the deletion of one gene (or the inhibition of its protein product) combined with the overexpression of a second gene results in cell death, while altering either gene alone is non-lethal [68,72,73,74]. Moreover, recent evidence indicates that the inverse relationship between PLK1 activity and HR is context-dependent rather than linear. Mechanistically, PLK1 can participate in the DDR by regulating RAD51 activation during HR-mediated repair, as shown by the PARP1–CHK1–PLK1–RAD51 axis in response to DNA damage [75]. Conversely, PLK1 overexpression has been reported to correlate with higher HRD scores, attenuated RAD51 foci formation and reduced HR efficiency, thereby increasing sensitivity to PARPi in cancer models [76,77]. Thus, PLK1 should not be presented simply as a kinase whose inhibition uniformly impairs HR. Rather, in DDR-impaired cells, PLK1 targeting is most defensible as a strategy to exploit reduced tolerance to replication-associated G2/M transition, mitotic entry and chromosome segregation under conditions of genomic imbalance [78,79]. These observations position PLK1 as a compelling candidate vulnerability in tumours with elevated replication-associated stress, including HR/ATM-deficient, ATR-impaired and Fanconi Anaemia pathway-defective backgrounds [80,81,82,83]. Nevertheless, PLK1 is also a broadly essential mitotic regulator, and its inhibition is not intrinsically specific to DDR-defective cells [84,85]. A more cautious interpretation is that DDR impairment may lower the tolerance threshold for PLK1 inhibition, particularly in tumours with high replication stress, defective checkpoint control or pre-existing CIN.

#### 3.1.3. Other Spindle Assembly Checkpoint (SAC)-Regulating Kinases

In addition to Aurora kinases and PLK1, other SAC regulators could be targeted to exploit potential vulnerabilities in genomically unstable cancers. The monopolar spindle Kinase 1 (MPS1 or TTK) kinase is a core SAC component essential for correct SAC activation and correction of erroneous kinetochore–microtubule attachments [86,87]. Therefore, an increased reliance on TTK/MPS1-mediated checkpoint surveillance may be observed upon broader DDR impairment due to the higher rate of mis-segregation events associated, for instance, with HRD status [53,86,88]. In this regard, TTK/MPS1 inhibition has been shown to induce catastrophic chromosome segregation in CIN-high cells [89,90,91,92,93] as well as HR impairment and radio-sensitisation in HR-proficient cells [94], although resistance due to point mutations in the TTK/MPS1 kinase domain may limit the therapeutic effects of these inhibitors [95,96]. Nevertheless, these data support the idea that SAC kinases may act as mitotic buffering nodes in CIN-high tumours, including some DDR-defective contexts (e.g., tumours with checkpoint impairment or *TP53* mutations). However, the strongest rationale for TTK/MPS1 inhibition relates to the CIN burden and checkpoint dependence rather than to the DDR mutation status alone, suggesting that biomarkers of chromosome mis-segregation or SAC reliance may be more informative than the HRD status in isolation [97,98].

Likewise, the serine/threonine kinase NIMA-related kinase 2 (NEK2), a pivotal regulator of centrosome separation and mitotic spindle organisation [99,100], has been implicated in mitotic stress tolerance [101], and its overexpression has been observed in tumours with elevated CIN [102,103,104]. Moreover, although direct mechanistic evidence linking NEK2 inhibition to HRD or other specific DDR gene mutations remains limited, NEK2 targeting has been shown to enhance the efficacy of CDK4/6 inhibitors and potentiate CIN induction [105]. Compared with Aurora kinases, PLK1 and TTK/MPS1, the link between NEK2 and DDR-defective tumour vulnerability remains less direct. Its inclusion in this context is mainly supported by its role in centrosome dynamics and CIN tolerance, rather than by strong evidence of selective lethality in HRD, ATM-deficient or replication checkpoint-defective models. NEK2 should therefore be considered an emerging candidate for further validation in CIN-high DDR-impaired tumours, rather than a validated synthetic lethal target.

### 3.2. Transcriptional and Cell-Cycle Regulatory Kinases

In highly proliferating cells, a critical source of genomic stress is represented by replication–transcription conflicts, which are further exacerbated in DDR-defective backgrounds, including HRD and ATM-impaired tumours [106,107]. Collisions between replication forks and actively transcribed regions can generate R-loops and DNA lesions, amplifying genomic instability. Moreover, the chronic activation of DDR pathways may cause alterations in transcriptional programs, promoting checkpoint adaptation and enabling cell-cycle progression despite the unresolved DNA damage [106,107,108]. Therefore, transcriptional and cell-cycle regulatory kinases may constitute critical nodes sustaining DDR-defective tumour survival by mitigating replication–transcription conflicts and maintaining stress-adaptive gene expression programs. In this scenario, DDR-defective tumours with high replication stress, CIN or replication-transcription conflicts may become more dependent on kinases regulating transcriptional elongation and cell-cycle progression, although the strength of this dependency is expected to vary across tumour contexts (Figure 2).

#### 3.2.1. Cyclin-Dependent Kinase 7 (CDK7)

As the catalytic subunit of CDK-activating kinase (CAK) and an essential component of the transcription factor II H (TFIIH), CDK7 activates other CDKs and regulates RNA polymerase II (Pol II) initiation and promoter clearance, thus playing critical roles in development and homeostasis by linking transcriptional control to cell-cycle progression [109]. Indeed, these CDK7 functions are central for cancer progression, as demonstrated by CDK7 hyperactivation and overexpression observed in multiple tumour types, where it predicts a poor prognosis and worse overall survival [109]. In this regard, selective CDK7 inhibitors, despite their cytostatic rather than cytotoxic effects, are gaining attention as promising therapeutic agents. In accordance with its roles, selective CDK7 inhibitors are currently being evaluated in clinical trials for multiple types of solid tumours, potentially offering alternative treatments for CDK4/6 inhibitors-resistant patients [109,110]. In HRD tumours, where replication stress and transcriptional pressure may be elevated, CDK7 inhibition could compromise stress-adaptive transcriptional programs, expression of DDR-associated survival genes and CDK-dependent cell-cycle progression [111]. However, the relationship between transcriptional inhibition, replication–transcription conflicts and therapeutic vulnerability is not necessarily unidirectional. Recent work showed that PARPi lethality in HR-deficient cells can be driven by transcription–replication conflicts and that inhibition of transcription elongation in the early S phase can reduce PARPi-induced damage and partially rescue HR-deficient cells from PARPi sensitivity [39]. Nevertheless, CDK7 inhibitors have demonstrated increased efficacy in transcriptionally addicted tumours [112], suggesting that DDR-defective tumours with marked transcriptional imbalance may represent a particularly relevant context [111]. Similarly, ATM-deficient backgrounds may display dysregulated transcriptional responses to DNA damage, potentially exhibiting an increased sensitivity to the disruption of stress-adaptive gene expression programs [113]. Therefore, CDK7 inhibition should not be assumed to synergise with HRD or PARPi solely by increasing replication–transcription conflicts. Its rational use in DDR-defective tumours is more likely to depend on demonstrated transcriptional addiction, persistent R-loop burden, stress-adaptive gene expression programs, residual checkpoint adaptation and treatment scheduling. Thus, CDK7 dependency should not be inferred from HRD or *ATM* loss alone, but should be evaluated in relation to the functional transcriptional stress state of the tumour.

#### 3.2.2. Cyclin-Dependent Kinase 9 (CDK9)

CDK9 regulates transcriptional elongation by phosphorylating the Pol II C-terminal domain, but also plays a direct regulatory role in transcription termination, making CDK9 a key regulator of transcription [114]. Relatedly, the dysregulation of the CDK9 pathway and its overexpression have been observed in multiple solid and haematological malignancies, where CDK9 is exploited by cancer cells to drive the expression of short-lived anti-apoptotic proteins like MCL-1 and Myc for their survival [115]. Indeed, in highly proliferative and genomically unstable cells, a sustained transcriptional activity is required to maintain the expression of different pro-survival players, including anti-apoptotic and DDR genes [116,117]. In this context, CDK9 inhibition may therefore attenuate stress-adaptive transcriptional programs [118,119], exacerbating the effects of DNA lesions’ accumulation in HR/DDR-defective cells. In agreement with this perspective, CDK9 inhibition has been reported to sensitise tumour cells to different DNA-damaging agents [120,121,122], further supporting its relevance as a potential genotoxic-stress sensitiser. Given that ATM and checkpoint dysfunctions alter transcriptional regulation following DNA damage [123,124,125], CDK9 targeting may be particularly relevant in DDR-impaired tumours that rely on short-lived pro-survival transcripts or stress-adaptive transcriptional programs. At present, however, CDK9 is better supported as a sensitiser to genotoxic stress than as a broadly validated DDR-defect-specific synthetic lethal target.

#### 3.2.3. Cyclin-Dependent Kinases 12 and 13 (CDK12/13)

CDK12 and CDK13 are both involved in the regulation of Pol II elongation as well as in the expression of long, multi-exonic genes, including several involved in DDR pathways and cell cycle control [126,127,128]. For this reason, CDK12 and CDK13 represent borderline/interface cases within the present framework: although they are not canonical DNA lesion sensors or repair enzymes, they directly influence DDR competence through transcriptional control of repair and cell-cycle genes. Their inclusion therefore reflects their role at the interface between transcriptional stress adaptation and genome maintenance, rather than their classification as canonical DDR targets. Nevertheless, while CDK12 has been extensively investigated for its role in sustaining the transcription of HR-associated genes [126,128], emerging evidence indicates that CDK13 may exert partially overlapping and non-redundant functions in transcriptional regulation and RNA processing [127]. Both kinases are involved in the correct expression of genes involved in DNA repair, replication and cell-cycle control, thereby contributing to the maintenance of genomic stability [127]. Genomic alterations of both CDK12 and CDK13 (including amplifications and truncating mutations) have been reported across different tumour types [129], where they contribute to cancer transcriptional addiction through aberrant gene expression programs that sustain cell proliferation and genomic instability [130]. However, both kinases appear to exert a dual role in cancer, resulting in different context-dependent phenotypes [126,130]: while their overexpression or amplification enhances oncogenic transcriptional programs, their loss-of-function impairs DDR gene expression promoting genomic instability [131,132]. In the context of DDR impairment, CDK12 and CDK13 play a critical role at the interface between transcriptional regulation and genome maintenance. CDK12 loss has been shown to induce a BRCAness-like phenotype characterised by reduced expression of HR genes and increased or restored sensitivity to PARPi [129,133,134,135]. Therefore, in tumours already harbouring DDR defects, including HRD or *ATM* loss, the pharmacological inhibition of CDK12/CDK13 could further impair the expression of residual DDR components and stress-response genes required to cope with persistent DNA damage, thereby exacerbating transcriptional dysregulation and genomic instability [129]. However, this logic should be interpreted cautiously, because CDK12 loss, CDK12 inhibition and pre-existing HRD may not produce equivalent biological or therapeutic outcomes across tumour types. Recent studies suggest that CDK13, similarly to CDK12, regulates RNA processing events, thus potentially contributing to transcriptional adaptation under stress conditions [136,137]. Since DDR-defective tumours frequently rely on compensatory transcriptional programs to sustain survival [106,107,108], simultaneously interfering with CDK12 and CDK13 activity may disrupt both transcriptional elongation and RNA maturation processes, thereby intensifying replication–transcription conflicts and compromising cell viability [129]. Thus, CDK12/CDK13 inhibition is best viewed as a strategy to perturb the transcriptional maintenance of genome stability and stress adaptation, with the strongest relevance in tumours whose survival depends on residual DDR gene expression or transcriptional buffering. The precise contribution of CDK13 to DDR-defective tumour vulnerability remains less defined compared to CDK12 and requires further validation.

#### 3.2.4. Dual-Specificity Tyrosine-Phosphorylation-Regulated Kinases (DYRKs)

In addition to classical CDKs, DYRK1A and DYRK1B have been implicated in the regulation of cell-cycle progression and stress adaptation [138,139]. By modulating transcription factors and cell-cycle regulators [140], DYRK1A/B can influence cell proliferation and checkpoint responses, showing a promising profile as a drug target in cancer cells as also demonstrated by recent evidence [140]. DYRK1B, although less extensively characterised, has been associated with the maintenance of cellular quiescence and survival under stress conditions, including hypoxia and nutrient deprivation, suggesting a potential role in promoting tumour cell adaptation to adverse microenvironments [141]. Despite the increasing effort in the development of DYRK1A/B inhibitors for cancer applications [141,142,143,144], direct evidence linking DYRK1A/B to DDR-defect-specific synthetic lethality remains limited [145]. Recent findings indicate a role for DYRK1A in DSB repair and HR-directed repair [140,146,147], whereas DYRK1B appears more closely associated with stress responses, quiescence and cell-cycle arrest [139,148,149]. Accordingly, DYRK kinases should be considered emerging regulators at the interface of cell-cycle control, transcriptional adaptation and stress tolerance. Their relevance to DDR-defective tumour vulnerability remains hypothesis-generating and requires validation in DDR-defined models.

### 3.3. Stress-Adaptation, Checkpoint and Survival Signalling Kinases

Besides replication and transcriptional perturbations, HRD as well as broader DDR impairment (such as *ATM* loss or ATR/CHK1 axis dysfunction) are associated with increased oxidative stress and the activation of adaptive signalling pathways [150,151,152,153]. ROS accumulation and metabolic imbalance may further damage DNA and proteins [48,153], making stress-response mechanisms pivotal for cellular viability maintenance [154]. This suggests that kinases involved in stress-response signalling, checkpoint adaptation and survival pathway rewiring may support tumour-cell tolerance of HR/DDR deficiency, although their relevance is likely to be highly context-dependent (Figure 2).

#### 3.3.1. Glycogen Synthase Kinase 3 Beta (GSK3β)

The multifunctional serine/threonine kinase GSK3β is involved in a plethora of diverse signalling pathways, including Wnt/β-catenin, phosphoinositide 3-kinase (PI3K)/Akt and mitochondrial regulation [155]. Due to its implication in a variety of diseases, including neurodegenerative disorders like Alzheimer’s disease [156,157] but also multiple tumour types [155], GSK3β has been extensively studied as a drug target in the past, as evidenced by the high number of selective inhibitors investigated either in clinical trials or at the preclinical level [158]. Among its diverse cellular functions, GSK3β also modulates cellular responses to DNA damage and oxidative stress [159], thereby influencing apoptosis-mediated cell death and mitochondrial function [160,161]. Recent evidence showed that GSK3β inhibition results in HR deficiency in colorectal cancer [162] and that GSK3β determines DDR pathway selection and tumour response to PARPi treatment in a BRCA1-independent manner [163], indicating an important interplay between GSK3β and DNA repair functions. Therefore, in HRD cellular contexts, characterised by persistent DNA lesions and metabolic alterations [42,43,46], GSK3β may contribute to sustaining adaptive responses like stress tolerance and survival signalling. In this regard, synthetic lethal interactions between GSK3β and fragile histidine triad protein (FHIT) in lung cancer were linked to an FHIT loss-induced abrogation of NHEJ and HR pathways [164], further supporting a pivotal role for GSK3β in DDR-compromised contexts. Interestingly, *ATM* loss has been linked to alterations of PI3K/Akt and GSK3β signalling dynamics [165,166], suggesting that GSK3β inhibition may exert context-dependent effects also in ATM-impaired tumours. Overall, GSK3β represents one of the more mechanistically interesting stress-adaptation kinases in DDR-impaired contexts, given its reported influence on HR efficiency, DDR pathway choice and PARPi response. However, because GSK3β has highly pleiotropic and context-dependent functions, its therapeutic relevance is unlikely to be defined by the DDR status alone and will require careful stratification according to the tumour lineage, repair state, metabolic context and survival signalling dependencies.

#### 3.3.2. Src Family Kinases (SFKs)

SFKs are cytosolic tyrosine kinases that integrate signals downstream of receptor tyrosine kinases, integrins and cytokine receptors to regulate cell proliferation, migration, cytoskeletal dynamics, and survival pathways [167,168]. In cancer cells, the aberrant activation of different SFK members has been associated with therapeutic resistance and signalling rewiring, suggesting that SFKs may function as central players enabling tumour cells to adapt to pharmacological stress [167]. In agreement with this perspective, Src activation observed following exposure to DNA-damaging agents may contribute to cell survival and resistance [169,170,171]. On the other hand, SFKs’ pharmacological inhibition has been shown to enhance the efficacy of different chemotherapeutic agents and targeted therapies in several tumour models [172,173], suggesting a role of SFK-mediated signalling in buffering stress-induced apoptosis. Furthermore, SFKs have been implicated in the regulation of cytoskeletal organisation and focal adhesion dynamics [174,175], processes that may influence mitotic fidelity and cellular responses to CIN [176,177]. Considering that HR/DDR deficiency imposes a chronic genotoxic burden and displays an increased reliance on adaptive signalling networks, Src, Fyn and other SFKs may then function as compensatory survival nodes against this background. In this regard, accumulating evidence indicates that Fyn and other SFKs may modulate cellular responses to genotoxic stress by regulating PI3K/Akt, MAPK and STAT signalling pathways [167], thus influencing apoptotic responses and cell-cycle progression. Although experimental evidence directly linking SFKs to canonical DNA repair mechanisms is limited, the available literature data suggest the existence of a cross-talk between SFKs and DDR pathways [178,179], where Src as well as other SFKs are required for the termination of ATR/CHK1 checkpoint signalling [180]. More recently, the SFK member Lck has been linked to HR-mediated repair activation in endometrial cancer by directly interacting and stabilising BRCA1 and RAD51 in response to DNA damage [181], potentially redefining Lck and other SFKs’ mechanistic impact in HR/DDR pathways’ regulation. Moreover, SFK inhibition may attenuate adaptive rewiring that sustains cell viability under genotoxic stress also in ATM-deficient or checkpoint-impaired tumours, where compensatory survival signalling is frequently activated [125]. Therefore, SFKs may contribute to adaptive survival signalling under genotoxic stress, including in HRD, ATM-deficient or checkpoint-impaired tumours where compensatory PI3K/Akt, MAPK or STAT signalling may sustain viability. However, SFKs should be interpreted primarily as mediators of pharmacological stress adaptation and signalling rewiring, rather than as validated DDR-defect-specific synthetic lethal targets. In addition, several clinically used SFK inhibitors are multi-kinase agents, making it necessary to distinguish SFK-dependent effects from broader off-target or polypharmacological activity.

#### 3.3.3. Proteotoxic, Checkpoint and Inflammatory Stress Adaptation Kinases

As a key mediator of the unfolded protein response (UPR) and the integrated stress response (ISR), protein kinase R (PKR)-like endoplasmic reticulum kinase (PERK) regulates translational attenuation and stress adaptation upon endoplasmic reticulum stress [44,182]. The rationale of targeting UPR components has been previously proposed as a strategy to selectively eliminate cancer cells with elevated proteotoxic burden [183]. Consistent with PERK being proposed as an important contributor to genomic instability, tumour microenvironment, aggressiveness, and chemoresistance adaptation [184], the increased protein synthesis demands and genomic instability peculiar to HRD tumours may lead to proteotoxic stress, resulting in PERK-dependent signalling activation. In this context, PERK inhibition may impair adaptive translational control and exacerbate stress-induced apoptosis in DDR-defective cells [185,186], including those with impaired ATM signalling, which has been implicated in mitochondrial and oxidative stress regulation [152]. Despite the limited number of studies linking PERK to DNA damage and repair, the available literature data indicate that PERK promotes cancer cell proliferation and tumour growth by limiting oxidative DNA damage [187] and inhibits DNA replication during UPR [188], suggesting the PERK-related pathway could modulate the response to DNA replication-targeted chemotherapies. Moreover, despite contrasting results reported in the literature [189], PERK has been implicated in radioresistance through interactions with the DDR machinery [190]. Thus, PERK may be relevant in DDR-defective tumours with high proteotoxic or oxidative burden, but the direct evidence for DDR-defect-specific dependency remains limited.

Among stress-response kinases, the p38 MAPK signalling downstream effector MAPK-activated protein kinase 2 (MAPKAPK2 or MK2) has a more direct rationale in checkpoint-defective settings. MK2 is a critical regulator of cell-cycle arrest in p53-deficient tumours [191], and synthetic lethal interactions have been reported between MK2 and p53 within DDR programs [192]. Since *TP53* mutations frequently co-occur with HRD and broader DDR impairment [193], reliance on MK2-mediated checkpoint adaptation may be enhanced in HR/DDR-compromised backgrounds. In this regard, MK2 inhibition has been reported to synergise with DNA-damaging agents in p53-deficient cells relying on ATM- and ATR-mediated checkpoint signalling through the p38 MAPK/MK2 pathway for survival [194], suggesting that stress-response kinases like MK2 may act as compensatory regulators when canonical checkpoint pathways are impaired.

The serine/threonine kinase TANK-binding kinase 1 (TBK1), mainly involved in innate immune signalling and cellular stress responses, has also been implicated in tumour survival under oncogenic and genotoxic stress [195]. Considering the increased micronuclei formation and cytosolic DNA sensing pathway activation observed with the HRD status [196], TBK1-mediated signalling may contribute to inflammatory and survival adaptation in DDR-compromised cells. This is supported by evidence linking TBK1 axis activation to PARPi resistance [197]. However, direct evidence that TBK1 inhibition selectively kills HRD or DDR-defective cells remains limited, and its role should therefore be framed as an emerging link between genome instability, innate immune signalling and therapy adaptation rather than as an established synthetic lethal vulnerability. Altogether, PERK, MK2 and TBK1 illustrate different ways in which DDR-defective tumours may engage stress-adaptation pathways: proteotoxic stress buffering, alternative checkpoint control and inflammatory survival signalling. Among these, MK2 currently has the clearest checkpoint-based rationale, whereas PERK and TBK1 remain more context-dependent and require stronger DDR-defined validation.

### 3.4. Metabolic and Redox-Related Kinases

Metabolic rewiring is increasingly recognised as a hallmark of cancer [198], and DNA repair processes are tightly connected with cellular energy homeostasis [199]. DDR-defective cells, including HRD and ATM-impaired contexts, characterised by persistent replication stress and ROS accumulation, may face increased energetic demands and redox imbalance [49,199], unveiling exploitable vulnerabilities with the context-dependent synthetic lethality approach (Figure 2). Noteworthily, although several central metabolic and survival pathways—such as PI3K/Akt and mechanistic target of rapamycin (mTOR) signalling cascades [200,201,202]—have been extensively investigated in cancer development and therapeutic resistance [203,204], their direct involvement in DNA repair and synthetic lethality strategies has been previously addressed and reviewed [13,205,206,207,208]. Hence, the present discussion focuses on less extensively characterised metabolic regulators that may act as context-dependent vulnerabilities in DDR-defective tumours, rather than revisiting canonical pathways whose role in oncogenic signalling and therapeutic targeting has already been comprehensively established. Taken together, metabolic and redox imbalance associated with DDR impairment may create additional dependencies on kinases regulating energy homeostasis and stress adaptation. However, compared with mitotic or transcriptional kinase dependencies, the evidence linking metabolic kinases to DDR-defect-specific vulnerability is generally less mature. These kinases should therefore be discussed as candidate modulators of stress tolerance whose therapeutic relevance will require stronger validation in DDR-defined models.

#### 3.4.1. AMP-Activated Protein Kinase (AMPK)

AMPK acts as a central energy sensor, coordinating metabolic adaptation under conditions of nutrient deprivation and stress [209]. In the cancer context, AMPK serves as metabolic master switch, typically acting as a tumour suppressor by inhibiting mTOR and protein synthesis under energy stress, but can also act as a contextual oncogene by enhancing the survival of nutrient-starved tumours [209,210,211]. In this regard, AMPK expression and activity levels vary among different cancer types, acting as both a therapeutic target and a factor in drug resistance [210,211]. As a consequence of the tight correlation between metabolism, cell cycle and DNA repair [199], AMPK plays a crucial role in maintaining genomic stability by directly enhancing specific DDR pathways [212] and oxidative stress-induced cell cycle arrest [213]. Under conditions of metabolic stress, activated AMPK promotes DNA repair through NHEJ and BER by phosphorylating key proteins like p53-binding protein 1 (53BP1) and wild-type p53-induced phosphatase 1 (WIP1) [214,215,216], favouring cell survival upon DNA damage accumulation. These observations suggest that HRD or DDR-compromised tumours may display an altered reliance on AMPK-dependent stress adaptation. However, the therapeutic directionality of AMPK modulation is not straightforward. AMPK can restrain anabolic growth and function as a tumour suppressor in some contexts, while supporting survival under energetic or oxidative stress in others. Therefore, whether AMPK inhibition or activation would be advantageous in DDR-defective tumours is likely to depend on the tumour lineage, metabolic state, ATM status and treatment context. Given that ATM directly regulates AMPK activation under oxidative stress [217] and that the ATM-AMPK-WIP1 feedback loop regulates DNA damage signalling and tumour stress responses [218], ATM-deficient tumours may display altered metabolic dependencies, but these should be considered context-specific rather than universally exploitable.

#### 3.4.2. Serum- and Glucocorticoid-Regulated Kinase 1 (SGK1) and Pyruvate Dehydrogenase Kinase (PDHK1)

Besides AMPK, other metabolic kinases could be considered within the context-dependent synthetic lethality rationale. The PI3K-adjacent kinase SGK1 has been implicated in cell survival, ion homeostasis and oxidative stress regulation [219]. Literature data suggest that SGK1 may compensate for Akt signalling under specific conditions [220,221] and has been reported to support survival in stress-adapted tumour cells [222]. Although limited, available data indicate that SGK1 cancer cell survival-promoting activity may be correlated with its role in DDR and genomic stability maintenance. Upon DNA damage, SGK1 phosphorylates HR essential components BRCA1 and RAD51, enhancing their activity [223] and suggesting a potentially increased reliance on SGK1 in DDR-compromised contexts. This is also supported by direct evidence linking SGK1 activation to the stress-response forkhead box O3 (FOXO3a)/p53 cross-talk upon DNA damage [224], further pointing at SGK1 as an interesting target in HR/DDR-deficient contexts. While direct evidence linking SGK1 inhibition to synthetic lethality in HRD is still lacking, its roles in oxidative stress regulation, survival signalling and reported modulation of BRCA1/RAD51 activity justify its consideration as an emerging adaptive kinase in DDR-impaired contexts. At present, however, SGK1 should be framed as a mechanistically plausible target rather than a validated DDR-defect-specific vulnerability.

Still within the metabolic/ROS context, PDHK1 regulates the pyruvate dehydrogenase complex, thereby controlling the pyruvate flux into the Krebs cycle [225,226,227]. By inhibiting the pyruvate dehydrogenase complex activity, PDHK1 promotes a shift towards glycolytic metabolism, thus contributing to the metabolic reprogramming frequently observed in cancer cells [225,227]. In DDR-defective contexts, where mitochondrial dysfunction and oxidative stress are often exacerbated [43], PDHK1-promoted metabolic adaptation may then support survival by limiting oxidative phosphorylation and reducing ROS production. In agreement, PDHK1 is implicated in metabolic plasticity regulation under stress conditions, including hypoxia and replication stress [225,227], which are commonly associated with DDR impairment. Noteworthily, an increased reliance on glycolytic pathways has been reported in genomically unstable tumours [43,228], suggesting that PDHK1-mediated metabolic rewiring may contribute to coping with the high oxidative and energetic stress levels usually observed in DDR-defective cells. Although direct evidence linking PDHK1 to DDR signalling or DDR-defect-specific synthetic lethality is currently lacking, its role in mitochondrial metabolism and redox balance suggests PDHK1 as a plausible candidate for further studies in stress-adapted DDR-impaired contexts. Its inclusion should therefore be viewed as hypothesis-generating rather than as evidence of an established kinase dependency.

## 4. Translating Non-Canonical DDR-Associated Kinase Vulnerabilities in DDR-Defective Tumours: Patient Stratification, Drug Combinations and Alternative Pharmacological Strategies

The kinase vulnerabilities discussed above differ substantially in their mechanistic basis, level of support and translational actionability. Some, such as mitotic kinases in CIN-high backgrounds or transcriptional kinases in replication–transcription conflict states, are supported by preclinical evidence of enhanced vulnerability or therapeutic sensitisation. Others, particularly metabolic and stress-adaptation kinases, remain more hypothesis-generating and require validation in DDR-defective models. Consequently, the clinical translation of therapies targeting non-canonical DDR-associated kinase vulnerabilities in DDR-defective tumours should be guided by the identification of the dominant stress phenotype, the rational selection of an appropriate pharmacological strategy, and effective management of treatment-associated toxicity and resistance mechanisms.

### 4.1. Patient Stratification: From DDR Genotype to Functional Stress Phenotypes

Patient stratification strategies based solely on genomic alterations may be insufficient to capture the functional state of DDR signalling. Although genomic alterations in DDR genes provide an initial framework for classification, they do not necessarily reflect current DNA repair capacity, replication fork protection, checkpoint rewiring or the adaptive kinase dependencies that emerge following DDR impairment. Likewise, genomic scar-based HRD scores (i.e., loss-of-heterozygosity, telomeric allelic imbalance, large-scale state transitions) capture the historical repair defects [229,230,231] but may not accurately represent ongoing replication stress, residual repair proficiency or kinase signalling at the time of treatment. Although HRD represents the most clinically validated paradigm, DDR dysfunction encompasses a broader spectrum of molecular alterations, including *BRCA1/2* or *PALB2* mutations, *ATM* loss, RAD51 dysfunction, DNA-PK alterations, replication checkpoint impairment, and TP53-associated checkpoint bypass. While mechanistically distinct, these lesions can generate partially overlapping stress phenotypes, including replication stress, CIN, replication–transcription conflicts, oxidative imbalance, proteotoxic stress and metabolic rewiring, which may create shared kinase dependencies. Therefore, patient stratification strategy should move from a single-layer DDR genotype model toward a functional stress-phenotype model. In this view, DDR alterations would represent an entry point rather than the final biomarker, with downstream stress phenotypes and associated kinase dependencies providing a more direct basis for identifying tumours most likely to benefit from targeting non-canonical DDR-associated kinases. This approach differs from conventional HRD scoring in both timing and biological resolution. Unlike genomic scar assays, functional stress phenotyping aims to capture the present state of tumour vulnerabilities, including dynamic changes induced by PARPi treatment, chemotherapy, radiotherapy or acquired resistance. This distinction is particularly relevant in tumours with HR restoration, fork stabilisation or treatment-induced signalling rewiring, where genomic HRD scars may persist despite altered therapeutic sensitivity.

From a practical perspective, the implementation of stress-phenotype-guided stratification would rely on a layered model in which DDR gene testing and genomic scar-based HRD assays provide an initial selection layer, while functional readouts are used as a second refinement layer to identify the dominant active stress phenotype. These readouts differ substantially in translational readiness. Genomic scar assays are already clinically implemented in selected tumour types, whereas RAD51 foci-based assays are closer to clinical implementation but still require broader standardisation of pre-analytical variables, scoring thresholds and inter-laboratory reproducibility. By contrast, DNA fibre assays, R-loop detection, phosphoproteomic profiling, metabolic flux measurements and several proteotoxic/oxidative stress readouts remain primarily research or early translational tools. Thus, the framework proposed here should be interpreted as a roadmap for biomarker development rather than as an immediately deployable clinical algorithm. Candidate readouts that may support this layered stress-phenotype-guided stratification strategy, together with their approximate translational readiness, are summarised in Table 1.

Importantly, this framework should not be interpreted as a clinically validated stratification algorithm. Most of these readouts remain preclinical, technically demanding or incompletely standardised. Rather, they define a translational roadmap for matching DDR-associated stress phenotypes to kinase dependencies. Clinical implementation will require a stepwise validation process, including (i) pre-analytical standardisation of sample collection, fixation, timing after treatment and assay conditions; (ii) harmonisation of scoring criteria and clinically meaningful thresholds; (iii) demonstration of inter-laboratory reproducibility; (iv) prospective evaluation of whether functional stress readouts predict kinase inhibitor response beyond established DDR gene alterations or HRD scores; and (v) assessment of how these readouts change under PARPi, chemotherapy, radiotherapy or acquired resistance. Such multidimensional approaches may help capture the temporal evolution of DDR-defective tumours, particularly under therapeutic pressure, where adaptive signalling networks may generate vulnerabilities that are not detectable at baseline. However, until such validation is achieved, functional stress phenotyping should be viewed as a refinement layer built on established genomic/scar-based biomarkers, rather than as a replacement for current patient-selection approaches.

Lineage-specific context represents an additional determinant that should be integrated with DDR genotype and functional stress phenotyping. The same DDR alteration may generate different kinase dependencies depending on tumour lineage, co-occurring alterations, baseline proliferation rate, transcriptional state, metabolic wiring, prior exposure to DNA-damaging therapies and tissue-specific tolerance to kinase inhibition. This consideration is particularly relevant because DDR-defective tumours should not be treated as a homogeneous group of HR-deficient cancers. While BRCA1/2-associated HRD remains the most clinically established model, other DDR-impaired contexts, including *ATM* loss, DNA-PK/NHEJ dysregulation, replication checkpoint impairment, TP53-associated checkpoint bypass and therapy-induced stress adaptation, may generate distinct combinations of replication stress, CIN, transcriptional imbalance, checkpoint reliance, oxidative/proteotoxic stress and metabolic rewiring. Therefore, stress-phenotype-guided kinase prioritisation should be interpreted in a lineage-aware manner (Table 2).

In addition to functional and lineage-aware stratification, translational prioritisation also requires distinguishing pharmacological tractability from clinical evidence in DDR-selected populations. Several agents targeting the kinase axes discussed in this review have entered oncology clinical testing, including Aurora kinase, PLK1, TTK/MPS1, CDK7, CDK9, GSK3β and SFK inhibitors. However, most available trials were conducted in tumour-type-defined or pathway-defined populations, such as ovarian cancer, acute myeloid leukaemia (AML), Kirsten rat sarcoma virus oncogene homologue (KRAS)-mutant colorectal cancer or hormone receptor-positive breast cancer, rather than in DDR-defective, HRD-selected or stress-phenotype-selected cohorts (Table A1, Appendix A). Therefore, clinical-stage development should be interpreted as evidence of druggability, not as proof of DDR-context clinical validation. Representative trial identifiers, patient populations and selected available outcomes for clinical-stage agents are provided in Appendix A (Table A1), while Table 3 summarises pharmacological tractability in relation to the kinase axes discussed in this review.

### 4.2. Limitations in Kinase Targeting Within DDR-Defective Contexts and Potential Overcoming Strategies

Despite the feasibility of pharmacologically targeting several non-canonical DDR-associated kinases, their translation in DDR-defective tumours may face both general and context-specific limitations. A major challenge is the high conservation of kinase domains, which can limit the selectivity and increase off-target effects due to scaffold similarity and kinase subtype cross-reactivity [242]. In addition, kinase inhibition is frequently associated with acquired resistance through kinase domain mutations, activation of compensatory signalling pathways or adaptive rewiring of signalling networks [242,243]. These issues may be particularly relevant in DDR-defective tumours, where genomic instability and treatment-induced selection can accelerate resistance evolution. Moreover, several constraints are especially important in this setting. First, DDR-associated kinase dependencies may be adaptive and dynamic rather than fixed (e.g., a kinase dispensable at baseline may become important after PARPi, chemotherapy or radiotherapy), whereas another dependency may disappear following HR restoration or replication fork stabilisation. Second, several kinases exert non-catalytic functions as scaffolding or signalling hubs, thus limiting the efficacy of ATP-competitive inhibitors that do not disrupt protein–protein interactions [244,245]. Third, many candidate kinases regulate essential processes such as mitosis, transcription, checkpoint control or energy homeostasis, raising concerns about the therapeutic window [246]. This is particularly relevant for combinations with PARPi, platinum agents or radiotherapy, where overlapping haematological, gastrointestinal or proliferative-tissue toxicities may limit dose intensity [247].

**Figure 3:**
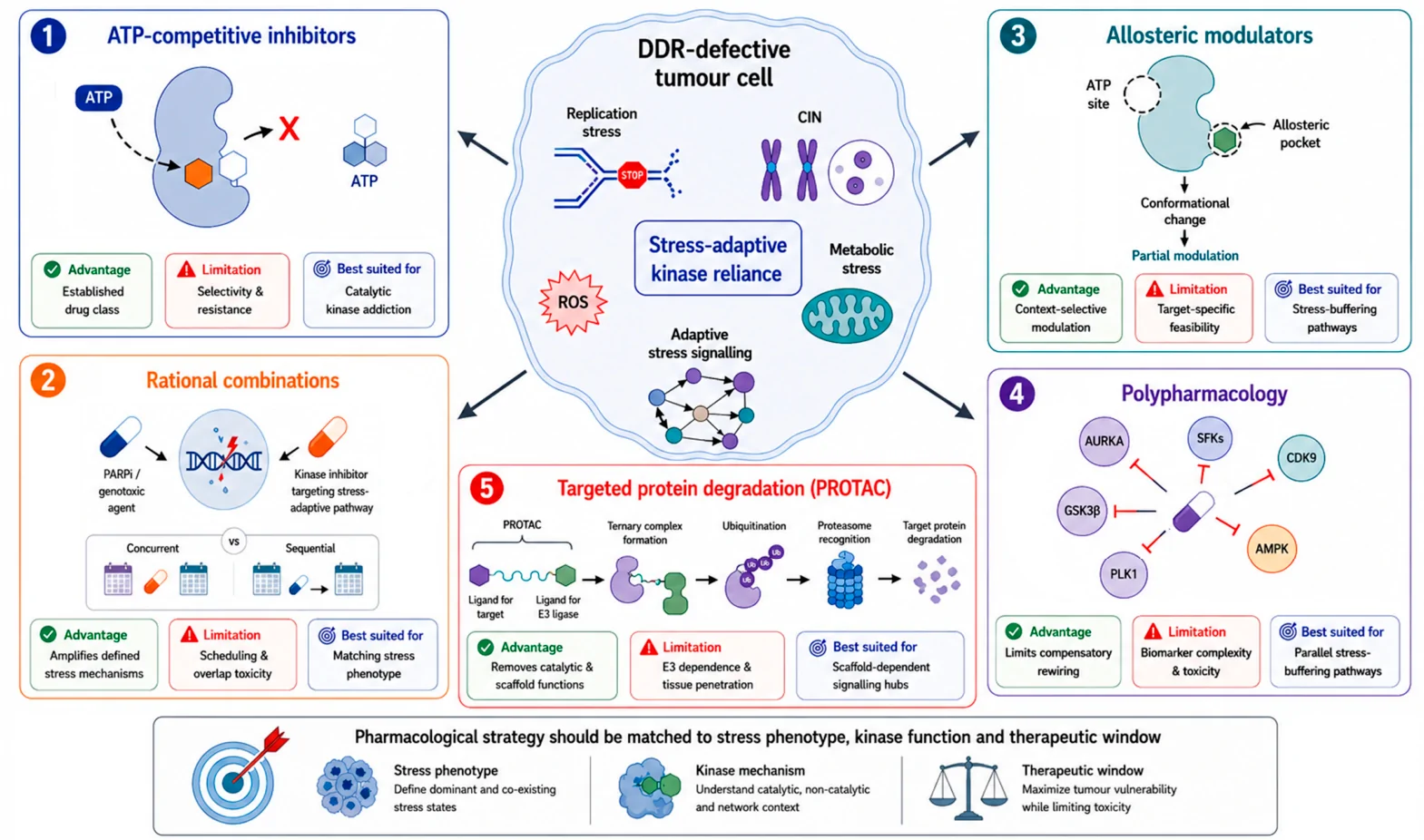
**Pharmacological strategies to target stress-adaptive kinase dependencies in DDR-defective tumours.** DDR-defective tumour cells may rely on stress-adaptive kinase signalling to tolerate replication stress, CIN, oxidative and metabolic stress, and adaptive survival pathway rewiring. Several pharmacological strategies can be used to perturb these dependencies. (1) ATP-competitive inhibitors block catalytic kinase activity and represent the most established modality, but may be limited by kinase-domain conservation, resistance mutations, incomplete disruption of non-catalytic functions and off-target effects. (2) Rational combinations, including PARPi or DNA-damaging agents with kinase inhibitors, may amplify defined stress mechanisms such as repair suppression, mitotic catastrophe, transcriptional collapse or stress-adaptation blockade; however, scheduling and overlapping toxicity are critical considerations. (3) Allosteric modulators may enable more context-selective or partial modulation of kinase activity, potentially exploiting the reduced stress tolerance of DDR-defective cells while preserving some physiological signalling. (4) Polypharmacological approaches may target parallel stress-buffering pathways and limit compensatory rewiring, but increase biomarker complexity and toxicity risk. (5) Proteolysis-targeting chimera (PROTAC)-mediated targeted protein degradation may remove both catalytic and scaffold functions of selected kinases, but remains dependent on E3 ligase expression, degradation kinetics, tumour penetration and tissue context. Overall, pharmacological targeting of non-canonical DDR-associated kinase vulnerabilities should be matched to the dominant stress phenotype, kinase mechanism, therapeutic window, treatment schedule and biomarker-defined tumour context.

Tumour selectivity should not be assumed based on a higher basal stress in DDR-defective cells. Several kinases discussed in this review (e.g., AURKA/B, PLK1, CDK7, CDK9 and CDK12/13) regulate core processes also required by normal proliferating cells, such as mitotic progression, transcriptional control, cell-cycle coordination and checkpoint adaptation. Their inhibition may therefore produce anti-tumour activity by general cytostatic or cytotoxic effects rather than through a DDR-context-specific vulnerability. In this setting, a therapeutic window is more likely to emerge when kinase dependency is quantitatively stronger in tumour cells than in normal tissues because the tumour is already close to a tolerability threshold for replication stress, CIN, transcriptional burden, checkpoint bypass, proteotoxic stress or metabolic imbalance. This implies that clinically useful targeting may require biomarker-defined dependency thresholds rather than binary DDR genotypes, together with schedules that separate stress induction from kinase inhibition. Intermittent, sequential or lower-dose regimens may be preferable to continuous maximal inhibition, particularly for combinations with PARPi, platinum agents or radiotherapy, where overlapping toxicities may otherwise obscure any synthetic lethal or sensitising effect. Thus, the feasibility of these strategies should be evaluated not only by tumour-cell killing, but also by whether a minimally effective and tolerable exposure can selectively exploit the dominant stress phenotype of DDR-defective tumour cells. These constraints highlight the need for strategies that account for the network-level and stress-dependent nature of DDR-associated vulnerabilities, rather than relying on indiscriminate single-kinase inhibition. The main pharmacological strategies that may be used to target non-canonical DDR-associated kinase vulnerabilities are summarised in Figure 3.

#### 4.2.1. Rational Combinations: Matching Kinase Inhibition to DDR-Associated Stress Mechanisms

Mounting preclinical evidence indicates that the inhibition of selected non-canonical DDR-associated kinases can potentiate PARPi-mediated cytotoxicity or sensitise tumour cells to DNA-damaging agents. In this regard, a first strategy is to combine PARPi or DNA-damaging agents with kinase inhibitors that further compromise residual DNA repair capacity. For instance, GSK3β inhibition has been reported to reduce HR efficiency and enhance PARPi-induced cell death, thereby mimicking a BRCAness-like phenotype in HR-proficient contexts [162,163]. Similarly, CDK12 inhibition may impair the transcription of HR-associated genes and enhance sensitivity to PARPi in both HRD and HR-proficient settings, with some evidence suggesting a role in overcoming resistance [248,249,250,251]. In these cases, the intended effect is not merely additive cytotoxicity, but deepening or inducing a BRCAness-like state. Nevertheless, this strategy requires careful patient selection, because tumours with complete HR loss, restored HR or fork-stabilising resistance mechanisms may respond differently.

A second strategy is to combine DDR-targeting agents with mitotic or SAC kinase inhibitors to convert unresolved DNA damage into mitotic catastrophe. AURKA inhibition has been shown to induce functional BRCAness [252], enhance PARPi sensitivity in both HRD and HR-proficient settings [253], and overcome PARPi resistance in selected models [254]. AURKB inhibition has also been proposed to sensitise tumour cells to PARPi-mediated cytotoxicity [255,256]. Likewise, PLK1 targeting has been associated with enhanced PARPi efficacy in non-HRD tumours [257], particularly under sequential inhibition strategies [258], and has been implicated in PARPi resistance reversal [259]. This rationale is strongest in tumours with high replication stress, micronuclei formation or pre-existing CIN, but may be limited by toxicity in proliferating normal tissues.

A third strategy is to suppress adaptive transcriptional, checkpoint or survival responses induced by genotoxic therapy. CDK9 inhibition has been reported to sensitise tumour cells to DNA-damaging agents and PARP inhibition, also in non-HRD contexts [120,260]. DYRK family members may also contribute to transcriptional regulation and stress adaptation, as DYRK1B inhibition has been shown to enhance PARPi sensitivity across HRD and non-HRD contexts [261]. In addition, SFK inhibition, including Src and Lck targeting, has been reported to enhance sensitivity to PARP inhibition in both HRD and non-HRD tumours [181,262,263]. For these kinases, the evidence often supports therapeutic sensitisation more strongly than direct DDR-defect-specific synthetic lethality. This point is particularly relevant for transcriptional kinase combinations, because dampening transcription may either suppress stress-adaptive gene expression or reduce transcription–replication conflicts depending on cell-cycle timing, treatment sequence and tumour context; therefore, such combinations should be guided by functional readouts such as R-loop burden, Pol II stress and S-phase γH2AX rather than inferred from the DDR genotype alone.

A fourth, more exploratory strategy is to combine DDR-targeting agents with modulators of oxidative, proteotoxic or metabolic stress adaptation. DDR-defective cells may display increased ROS production, mitochondrial dysfunction and altered energetic demands, potentially creating sensitivity to perturbation of PERK, AMPK, SGK1 or PDHK1 signalling. However, this area remains less substantiated than mitotic or transcriptional kinase targeting, and therapeutic actionability may be context-dependent, particularly for AMPK. Regardless of the employed strategy, these combinations may maximise stress accumulation but also increase toxicity, whereas sequential treatment may allow DDR-targeting agents to induce replication stress before mitotic, transcriptional or stress-adaptation dependencies are therapeutically exploited. Hence, the optimal schedule may vary—according to whether the goal is repair suppression, mitotic catastrophe, transcriptional collapse or stress-adaptation blockade—and remains to be systematically investigated. Importantly, such scheduling should also be considered a therapeutic-window strategy, with the aim of identifying exposure levels and treatment sequences that are sufficient to exploit tumour-specific stress dependency while limiting prolonged suppression of essential kinase functions in normal proliferating tissues.

#### 4.2.2. Kinase Allosteric Modulation and Polypharmacology Strategies

In DDR-defective tumours, kinase targeting does not necessarily require complete pathway suppression. Because these tumours may already face near thresholds of replication stress, CIN, transcriptional imbalance or oxidative stress, partial or context-selective disruption of adaptive kinase functions may be sufficient to induce therapeutic vulnerability. This provides a rationale for allosteric modulation, which may alter kinase conformation, substrate engagement or regulatory interactions without directly competing with ATP binding, potentially improving selectivity and reducing off-target toxicity [264]. Several examples are relevant to the kinase axes discussed above. GSK3β represents a prototypical example where allosteric modulation may allow context-dependent inhibition while preserving physiological signalling [155].

AURKA allosteric modulation has been proposed as a strategy to disrupt spindle assembly functions without fully inhibiting catalytic activity, potentially reducing toxicity associated with complete kinase blockade [265]. Similarly, allosteric inhibitors of TTK/MPS1 and PLK1 have been described, targeting regulatory domains involved in kinase activation and substrate recognition rather than ATP binding [266,267]. Such approaches may be particularly relevant in DDR-defective tumours, where partial disruption of mitotic checkpoint or CIN-tolerance functions may be sufficient to push beyond tolerable thresholds. Beyond mitotic kinases, allosteric regulation has also been explored in metabolic and stress-related pathways. AMPK allosteric modulators have been developed to fine-tune energy sensing and metabolic adaptation [268], whereas allosteric targeting of transcriptional kinases such as CDK12/13 or regulatory interfaces within SFKs may provide opportunities to disrupt catalytic and non-catalytic functions involved in stress adaptation [269,270]. Nevertheless, allosteric approaches remain unevenly developed across the kinases discussed here, and for many targets, the availability of potent, selective and clinically suitable allosteric modulators remains limited. Their value in DDR-defective tumours will need to be demonstrated functionally rather than assumed from improved selectivity alone.

Besides combinatorial and single-agent approaches, polypharmacology may be useful where DDR-defective tumours rely on parallel stress-buffering pathways. Multi-kinase inhibitors, including dual inhibitors targeting pathway-related kinases [271] or compounds directed against kinases involved in different survival nodes (e.g., a recent example of triple kinase inhibitor targeting GSK3β, Fyn and DYRK1A [272]), may reduce compensatory pathway activation and limit the emergence of resistance. However, multi-target inhibition also complicates biomarker selection, mechanism attribution and toxicity management. Therefore, polypharmacological strategies should be justified by defined stress phenotypes and pathway co-dependencies, not by broad anti-cancer activity alone.

#### 4.2.3. Targeting Non-Canonical DDR-Associated Kinases Through Proteolysis-Targeting Chimeras (PROTACs)

PROTACs are bifunctional molecules that simultaneously bind an E3 ubiquitin ligase and a target protein to promote its ubiquitination and proteasomal degradation [273]. Unlike ATP-competitive inhibitors, PROTACs enable a selective removal of target kinase, thereby disrupting both catalytic and non-catalytic functions of kinases, including scaffolding and signalling roles contributing to stress tolerance and adaptive rewiring in DDR-defective tumours [273,274,275]. This distinction may be important for kinases involved in mitotic complexes, transcriptional regulation or survival signalling hubs, where complete degradation may more effectively disrupt network-level dependencies compared to partial enzymatic inhibition [276,277,278].

Targeted degradation may help address selected limitations of conventional kinase inhibition, e.g., by mitigating some resistance mechanisms linked to kinase-domain mutations or by disrupting non-catalytic scaffolding functions [277,278]. However, degradation is not intrinsically more selective or less toxic than inhibition; its advantage must be demonstrated for each target and tumour context. Notably, increasing efforts have been directed towards the development of PROTACs targeting kinases involved in stress buffering across multiple functional axes. Mitotic kinases such as AURKA, AURKB, PLK1 and TTK/MPS1 have been successfully targeted by degradation-based approaches, demonstrating efficient protein depletion and disruption of mitotic progression [279,280,281,282,283,284,285]. Transcriptional kinases, including CDK7, CDK9 and CDK12/13, have also been targeted, with degradation leading to suppression of transcriptional programs and, in some cases, enhanced sensitivity to PARP inhibition [133,286,287,288,289,290]. Degradation strategies have further been extended to kinases involved in stress adaptation and signalling plasticity, including SFKs (including Src and Lck), DYRK family members, GSK3β and TBK1 [291,292,293,294,295,296]. Emerging efforts to target metabolic regulators such as PDHK1 further highlight the versatility of degradation-based approaches [297]. These examples demonstrate technical feasibility, but not necessarily DDR-defective clinical relevance.

In DDR-defective tumours, targeted degradation could be useful when the complete removal of a stress-buffering kinase results in a prolonged collapse of adaptive signalling compared with catalytic inhibition. Evidence that PROTAC-mediated degradation of transcriptional CDKs may enhance sensitivity to PARP inhibition supports the possibility that degradation-based strategies may complement conventional inhibitors in selected combinatorial settings. Nevertheless, PROTACs introduce additional translational constraints, including dependence on E3 ligase expression, tumour penetration, ternary-complex formation, degradation kinetics and tissue-specific proteostasis capacity. For essential kinases such as PLK1, CDK7 or CDK9, complete degradation may increase toxicity compared with partial enzymatic inhibition. Moreover, few kinase degraders have been validated specifically in DDR-defective or HRD-selected models. Therefore, targeted degradation should be viewed as a promising but still largely preclinical strategy whose value will depend on demonstrating an improved selectivity, therapeutic window or resistance suppression over conventional inhibition.

Overall, the translational implementation of non-canonical DDR-associated kinase targeting will depend on matching the pharmacological strategy to the biology of the dominant stress phenotype. Mitotic kinase inhibition is most rational in CIN-high or replication-stress-high tumours; transcriptional kinase targeting may be most relevant where replication–transcription conflicts or stress-adaptive transcriptional programs are evident; checkpoint and survival signalling kinases may be prioritised in TP53-deficient or therapy-adapted states; and metabolic kinase targeting remains exploratory unless supported by functional evidence of redox or energetic dependency. This stress-phenotype-guided approach may help avoid indiscriminate kinase combinations and prioritise contexts in which synthetic lethality, therapeutic sensitisation or stress-adaptive vulnerability is biologically plausible.

## 5. Conclusions and Future Directions

DDR impairment, whether arising from HRD, *ATM* loss, replication checkpoint dysfunction, DNA-PK/NHEJ dysregulation or other repair defects, should not be viewed only as defective DNA repair. Instead, these alterations generate interconnected stress phenotypes, including replication fork instability, CIN, transcriptional and cell-cycle imbalance, oxidative/proteotoxic stress and metabolic rewiring, that collectively reshape tumour-cell dependencies. Within this framework, non-canonical DDR-associated kinases linked to these stress phenotypes may function as adaptive survival nodes, enabling and fostering tumour cells’ survival and drug resistance.

However, the therapeutic relevance of these kinases is not uniform, varying substantially across functional categories and tumour contexts. Mitotic and transcriptional kinases are currently supported by stronger mechanistic or preclinical evidence in settings of CIN, replication stress, replication–transcription conflicts or PARPi sensitisation, whereas several stress-adaptation and metabolic kinases remain more exploratory. Therefore, their prioritisation should be guided by the underlying DDR lesion, dominant stress phenotype, tumour lineage, treatment history, therapeutic window and strength of supporting evidence, rather than by the DDR genotype alone. Consequently, effective exploitation of these vulnerabilities will require mechanism-guided approaches integrating rational combinations, careful treatment scheduling, allosteric modulation, polypharmacology or targeted protein degradation where biologically justified.

Future development of this framework will require prospective validation in models defined by the DDR status and stress phenotype, together with biomarker strategies that integrate DNA repair competence, replication fork stability, CIN, checkpoint adaptation, transcriptional stress, phosphoproteomic signalling and the metabolic state. Importantly, these dependencies should also be considered dynamic, since PARPi, chemotherapy, radiotherapy or acquired resistance may induce new vulnerabilities not detectable at baseline. Overall, stress-phenotype-guided kinase targeting may refine the synthetic lethality paradigm and expand therapeutic opportunities in DDR-defective malignancies, provided that candidate vulnerabilities are functionally validated, clinically contextualised and matched to tolerable pharmacological strategies.

## Figures and Tables

**Table 1:** **Functional stress phenotypes, candidate biomarkers and corresponding kinase dependencies in DDR-defective tumours.** Translational-readiness codes are reported in square brackets after each candidate biomarker/readout and are defined in the table notes.

Functional Stress Phenotype	Candidate Biomarker/Readout	Candidate Kinase Axis	Therapeutic Implication
Impaired HR competence	Reduced RAD51 foci formation [**N**]; BRCA1/2, PALB2 or RAD51 paralog alterations [**C**]; genomic scar signatures [**C**]	AURKA/B, CDK12/13, GSK3β	Identify tumours with persistent or inducible repair vulnerability
Replication fork instability	DNA fibre assays [**P**]; fork degradation [**P**]; phospho-RPA [**T**]; S-phase γH2AX foci [**T**]	PLK1, AURKA, CDK7, CDK9	Impaired tolerance to replication stress, aberrant mitotic entry or transcriptional-associated stress
CIN-high state	Micronuclei [**T**/**P**]; anaphase bridges [**T**/**P**]; lagging chromosomes [**T**/**P**]; copy-number complexity [**C**/**T**]; CIN signatures [**T**]	AURKA/B, PLK1, TTK/MPS1, NEK2	Prioritise mitotic and SAC-targeting strategies
Replication–transcription conflict	R-loop markers [**P**]; Rnase H-sensitive damage [**P**]; Pol II elongation stress [**P**]; γH2AX at transcribed loci [**P**]	CDK7, CDK9, CDK12/13, DYRKs	Target transcriptional stress-adaptive gene expression
TP53 mutation or loss/checkpoint adaptation	TP53 mutation [**C**]; p38/MK2 activation [**T**/**P**]; G2/M checkpoint dependence [**P**]	MK2, PLK1, TTK/MPS1	Exploit reliance on alternative checkpoint mechanisms
Oxidative/proteotoxic stress	ROS markers [**T**/**P**]; NRF2, ISR or UPR activation [**T**/**P**]; PERK-eIF2α signalling [**P**/**E**]	PERK, GSK3β, SFKs, TBK1	Target stress-response signalling where dependency is functionally demonstrated
Metabolic rewiring	AMPK activation [**T**/**P**]; mitochondrial dysfunction [**P**/**E**]; glycolytic reprogramming [**P**/**E**]; altered pyruvate metabolism [**P**/**E**]; redox imbalance [**P**/**E**]	AMPK, SGK1, PDHK1	Consider metabolic kinase targeting only when energetic or redox dependency is functionally supported

Notes: The kinase dependencies reported here are not restricted to a single stress phenotype; multiple DDR impairment-associated stress phenotypes may converge on kinases shared by different signalling pathways. The indicated translational readiness refers to the approximate maturity of the biomarker/readout, not to clinical validation of the corresponding kinase-targeting strategy. **Readiness codes**: **C**, clinically implemented in selected tumour contexts; **N**, near-clinical or undergoing broader standardisation; **T**, translationally adaptable but not routine; **P**, primarily preclinical/research use; **E**, exploratory or context-specific. Readiness codes refer to the approximate maturity of the biomarker/readout, not to clinical validation of the corresponding kinase-targeting strategy. All abbreviations used in the table are defined in the Abbreviations section.

**Table 2:** **Lineage-specific determinants that may influence non-canonical DDR-associated kinase dependencies.** Representative tumour contexts are shown to illustrate how DDR lesion type, co-mutational background and dominant stress phenotypes may shape kinase-dependency prioritisation. These associations should be interpreted as a framework for functional validation rather than as treatment-selection rules.

Tumour Context	Dominant DDR/Stress Features	Potentially Informative Kinase Axes	Main Caveat	Refs.
High-grade serous ovarian cancer	Frequent HRD/BRCAness, near-universal TP53 mutation, high CIN, platinum/PARPi exposure and resistance	AURKA/B, PLK1, TTK/MPS1; CDK12/13; GSK3β	HRD scars may persist despite HR restoration, fork stabilisation or treatment-induced rewiring	[232,233]
Triple-negative breast cancer	BRCA1/2-mutated or BRCAness-like subsets, high proliferation, replication stress, CIN, frequent TP53 mutation	AURKA/B, PLK1, TTK/MPS1; CDK7/CDK9; MK2	BRCAness is heterogeneous; HR impairment, CIN and transcriptional stress require functional discrimination	[234,235]
Prostate cancer	BRCA2, ATM and CDK12 alterations; AR-driven transcriptional programs; lineage plasticity	CDK12/13, CDK7/CDK9; GSK3β; PLK1	BRCA2, ATM and CDK12 alterations are not functionally equivalent and may predict different dependencies	[236,237]
Pancreatic cancer	BRCA1/2 or PALB2-mutant subset, ATM alterations, KRAS-driven signalling, hypoxia and metabolic stress	GSK3β, SFKs; PERK/ISR; AMPK/PDHK1; CDK7/CDK9	HRD-positive cases are a minority; metabolic/stress-adaptation axes remain largely hypothesis-generating	[238,239]
Lung cancer	*ATM* loss, TP53 co-alterations, oxidative stress, replication stress, FHIT loss in selected contexts, oncogenic signalling rewiring	GSK3β; SFKs; MK2; PLK1/AURKA in CIN-high states	DDR lesions are heterogeneous and often coexist with dominant oncogenic signalling programs	[124,164]
Haematological malignancies	High proliferative pressure, replication stress, transcriptional addiction, variable TP53/ATM defects	CDK7/CDK9; PLK1; AURKA/B; MK2	Therapeutic window may be limited by myelosuppression and toxicity in normal haematopoiesis	[240,241]

Note: Tumour contexts are representative rather than exhaustive. The indicated kinase axes do not represent lineage-specific therapeutic recommendations, but examples of how DDR alteration type, co-mutational background and dominant stress phenotype may guide functional validation. All abbreviations used in the table are defined in the Abbreviations section.

**Table 3:** **Representative pharmacological tractability for selected non-canonical DDR-associated kinase axes discussed in this review.**

Kinase	Representative Agent(s)	Modality	Development Status/Representative Clinical Context	DDR-Defective Context Rationale
AURKA	Alisertib/MLN8237	ATP-competitive inhibitors	Clinical stage; evaluated in recurrent or platinum-resistant/refractory ovarian cancer and other oncology settings	Relevant to CIN-high and DDR-impaired contexts; evidence includes functional BRCAness, PARPi sensitisation and mitotic vulnerability
AURKB	Barasertib/AZD1152	ATP-competitive inhibitors	Clinical stage; mainly evaluated in AML	Potentially relevant where DDR defects increase chromosome mis-segregation; DDR-specific evidence is less mature than general mitotic-toxicity rationale
PLK1	Volasertib; onvansertib	ATP-competitive inhibitors	Clinical stage; volasertib mainly in AML, onvansertib in KRAS-mutant metastatic colorectal cancer combinations	Relevant to replication-stress/CIN-high DDR-defective tumours; sequential PARPi combinations may be important
TTK/MPS1	CFI-402257; BAY 1161909	ATP-competitive inhibitors	Early clinical; evaluated in advanced solid tumours, HER2-negative breast cancer and paclitaxel-combination settings	Most relevant in CIN-high tumours; DDR genotype alone may be insufficient for selection
NEK2	NEK2 inhibitors	Small-molecule inhibitors	Preclinical	Mainly mechanistic rationale; limited DDR-defined validation
CDK7	Samuraciclib; SY-5609	ATP-competitive inhibitors	Clinical stage; evaluated in advanced solid tumours, breast cancer and pancreatic cancer-oriented development	Potentially relevant in replication–transcription conflict and transcriptionally addicted DDR-impaired tumours
CDK9	Alvocidib; AZD4573	ATP-competitive inhibitors	Clinical stage; mainly evaluated in AML and other haematological malignancies, including MCL-1/apoptotic-dependency contexts	Better supported as a genotoxic/PARPi sensitiser than as a direct DDR-defect-specific synthetic lethal target
CDK12/CDK13	THZ531-like compounds; selective or dual CDK12/13 inhibitors; CDK12/13 degraders	Covalent inhibitors/selective inhibitors/degraders	Preclinical	Strong DDR-transcription interface, but CDK12 loss, pharmacological inhibition and HRD are not equivalent
DYRK1A/DYRK1B	Harmine derivatives; EHT1610-related inhibitors; other DYRK inhibitors	Small-molecule inhibitors	Preclinical	Hypothesis-generating for DDR-defective tumours; stronger validation in DDR-defined models is needed
GSK3β	LY2090314; tideglusib; other GSK3β inhibitors/allosteric modulators	ATP-competitive and allosteric inhibitors	Early clinical/non-oncology development; LY2090314 evaluated in advanced cancer and pancreatic cancer combinations; tideglusib mainly non-oncology development	DDR relevance supported by HR modulation, pathway-choice effects and PARPi response links, but highly context-dependent
SFKs	Dasatinib; bosutinib	Multi-kinase inhibitors	Approved in Ph+ haematological malignancies	May attenuate genotoxic-stress survival signalling and PARPi resistance; target attribution complicated by multi-kinase activity
PERK	GSK2656157; AMG44	Small-molecule inhibitors	Preclinical/early development	Rationale in proteotoxic/oxidative stress-high DDR-defective tumours; DDR-specific dependency remains limited
MK2	PF-3644022; other MK2 inhibitors	Small-molecule inhibitors/tool compounds	Preclinical/tool-compound stage	Particularly relevant to TP53-deficient checkpoint adaptation and genotoxic-stress sensitisation
TBK1	BX795-like compounds; MRT67307-like compounds; emerging TBK1 degraders	Small-molecule inhibitors/degraders	Preclinical	Emerging link to micronuclei/cytosolic DNA signalling and PARPi resistance; HRD-specific killing is not established
AMPK	Metformin; A-769662; other AMPK modulators	Indirect activator/allosteric modulators	Metformin approved for metabolic disease and widely explored in oncology; direct AMPK modulators mostly preclinical	ATM–AMPK crosstalk and metabolic adaptation are relevant, but therapeutic directionality is context-dependent
SGK1	GSK650394; other SGK inhibitors	Small-molecule inhibitors	Preclinical	Mechanistically plausible through oxidative stress and BRCA1/RAD51 modulation; direct DDR-defective vulnerability remains limited
PDHK1	Dichloroacetate; AZD7545	Metabolic enzyme inhibitors	Preclinical/repurposing-oriented	Hypothesis-generating in redox/metabolic stress-adapted DDR-defective tumours; DDR-specific validation is lacking

Notes: This table is intended as a selective overview of pharmacological tractability rather than a comprehensive catalogue of inhibitors. Development status and representative clinical contexts refer to oncology studies of selected compounds or compound classes and do not necessarily indicate clinical testing in DDR-defective, HRD-selected or stress-phenotype-selected populations. DDR-context relevance summarises the rationale discussed in this review and should not be interpreted as evidence of clinical efficacy in DDR-defective tumours. All abbreviations used in the table are defined in the Abbreviations section.

## Data Availability

No new data were created or analyzed in this study. Data sharing is not applicable to this article.

## Abbreviations

The following abbreviations are used in this manuscript:

53BP1	p53-binding protein 1
Akt	Protein kinase B
AML	Acute myeloid leukaemia
AMPK	AMP-activated protein kinase
alt-EJ	Alternative end joining
ATM	Ataxia-telangiectasia mutated
ATR	Ataxia-telangiectasia and Rad3-related protein
AURKA	Aurora kinase A
AURKB	Aurora kinase B
BARD1	BRCA1-associated RING domain protein 1
BER	Base excision repair
BRCA	Breast cancer susceptibility gene/protein
CAK	CDK-activating kinase
CDK	Cyclin-dependent kinase
CHK1	Checkpoint kinase 1
CIN	Chromosomal instability
DDR	DNA damage response
DNA-PK	DNA-dependent protein kinase
DSB	DNA double-strand break
DYRK1A	Dual-specificity tyrosine-phosphorylation-regulated kinase 1A
DYRK1B	Dual-specificity tyrosine-phosphorylation-regulated kinase 1B
E3	E3 ubiquitin ligase
eIF2α	Eukaryotic translation initiation factor 2 alpha
EIF2AK3	Eukaryotic translation initiation factor 2 alpha kinase 3; gene name for PERK
FANCA	Fanconi anaemia complementation group A
FHIT	Fragile histidine triad protein
FOXO3a	Forkhead box O3
Fyn	Fyn-related Src family tyrosine kinase
G2	Gap 2 phase of the cell cycle
G2/M	Gap 2/mitosis transition
GSK3β	Glycogen synthase kinase 3 beta
H2AX	H2A histone family member X
γH2AX	Phosphorylated H2AX
HR	Homologous recombination
HRD	Homologous recombination deficiency
ISR	Integrated stress response
KRAS	Kirsten rat sarcoma virus oncogene homologue
Lck	Lymphocyte-specific protein tyrosine kinase
MAPK	Mitogen-activated protein kinase
MAPKAPK2	Mitogen-activated protein kinase-activated protein kinase 2
MCL-1	Myeloid cell leukaemia 1
MK2	MAP kinase-activated protein kinase 2
MPS1	Monopolar spindle 1 kinase
mTOR	Mechanistic target of rapamycin
MRE11	Double-strand break repair protein meiotic recombination 11
Myc	Myc proto-oncogene/transcription factor
NEK2	NIMA-related kinase 2
NER	Nucleotide excision repair
NHEJ	Non-homologous end joining
NIMA	Never in mitosis A
NRF2	Nuclear factor erythroid 2-related factor 2
PALB2	Partner and localiser of BRCA2
PARP	Poly(ADP-ribose) polymerase
PARPi	PARP inhibitor
PDHK1	Pyruvate dehydrogenase kinase 1
PERK	protein kinase R (PKR)-like endoplasmic reticulum kinase
PI3K	Phosphoinositide 3-kinase
PLK1	Polo-like kinase 1
Pol II	RNA polymerase II
PROTAC	Proteolysis-targeting chimera
p38 MAPK	p38 mitogen-activated protein kinase
p53	Protein product of TP53
p-RPA	Phosphorylated replication protein A
RAD51	Radiation sensitive protein 51/RAD51 recombinase
RAD51B	RAD51 paralog B
RAD51C	RAD51 paralog C
RAD51D	RAD51 paralog D
R-loops	RNA–DNA hybrid structures with displaced single-stranded DNA
ROS	Reactive oxygen species
RPA	Replication protein A
SAC	Spindle assembly checkpoint
SFKs	Src family kinases
SGK1	Serum- and glucocorticoid-regulated kinase 1
Src	SRC proto-oncogene tyrosine-protein kinase
SSA	Single-strand annealing
SSB	DNA single-strand break
STAT	Signal transducer and activator of transcription
TBK1	TANK-binding kinase 1
TFIIH	Transcription factor II H
TP53	Tumour protein p53 gene
TTK/MPS1	TTK protein kinase/monopolar spindle 1 kinase
UPR	Unfolded protein response
WEE1	Wee1 G2 checkpoint kinase
WIP1	Wild-type p53-induced phosphatase 1
XRCC2	X-ray repair cross-complementing protein 2
XRCC3	X-ray repair cross-complementing protein 3

## Appendix A

To complement the pharmacological tractability overview reported in Table 3, Table A1 summarises representative clinical-trial contexts for selected approved or clinical-stage inhibitors targeting kinase axes discussed in this review. The table reports trial identifiers, patient populations or selection contexts, and selected available outcomes or development status, with the aim of clarifying the type of clinical evidence underlying the “clinical-stage” designation. Importantly, these trials were generally conducted in tumour-type-defined or pathway-defined populations rather than in DDR-defective, HRD-selected or stress-phenotype-selected cohorts; therefore, they should be interpreted as evidence of pharmacological and clinical feasibility, not as proof of DDR-context-specific clinical efficacy.

**Table A1:** Representative clinical-trial contexts for selected clinical-stage inhibitors targeting kinase axes discussed in Table 3.

Kinase Axis/Representative Agent	Representative Clinical Trial(s)	Patient Population/Clinical Context	Selected Available Outcome or Status	DDR-/HRD-Selected Clinical Validation
AURKA—alisertib/MLN8237	NCT00853307; NCT01091428	Platinum-resistant or platinum-refractory epithelial ovarian, fallopian tube or primary peritoneal carcinoma; recurrent ovarian cancer ± prior breast-cancer phase I component	Single-agent alisertib produced stable disease in 52% of patients in a phase II platinum-resistant/refractory ovarian study; alisertib plus weekly paclitaxel significantly favoured progression-free survival versus paclitaxel alone in recurrent ovarian cancer.	Not primarily DDR-/HRD-selected; ovarian cancer context is DDR-relevant but trial selection was not based on HRD/stress phenotype.
AURKB—barasertib/AZD1152	NCT00497991; NCT00926731; NCT00952588	Mainly newly diagnosed, relapsed/refractory or older/unfit AML populations	Phase I/II AML data reported manageable toxicity and a 25% overall haematologic response rate; barasertib plus low-dose cytarabine reported a 45% overall response rate in an elderly AML phase I study.	Not DDR-selected; clinical context mainly AML/mitotic kinase targeting.
PLK1—volasertib	NCT01721876	Older patients with previously untreated AML ineligible for intensive induction therapy	Phase III POLO-AML-2 evaluated volasertib plus low-dose cytarabine versus placebo plus low-dose cytarabine in this population.	Not DDR-selected; AML development rather than DDR-/HRD-stratified clinical testing.
PLK1—onvansertib	NCT03829410; NCT06106308	KRAS-mutant metastatic colorectal cancer, including second-line FOLFIRI plus bevacizumab combinations	Phase Ib/II studies evaluated onvansertib with FOLFIRI/bevacizumab in KRAS-mutant metastatic colorectal cancer after prior oxaliplatin exposure.	Not DDR-selected; RAS-mutant colorectal cancer setting.
TTK/MPS1—CFI-402257	NCT02792465; NCT03568422	Advanced solid tumours and breast cancer, including HER2-negative metastatic breast cancer combinations	Early-phase studies evaluated CFI-402257 alone or with paclitaxel/fulvestrant; reported breast-cancer activity remains limited and early-stage.	Not DDR-selected; clinical development is based on mitotic/SAC targeting rather than DDR stratification.
TTK/MPS1—BAY 1161909	NCT02138812	Advanced solid malignancies, including combination with paclitaxel	Early clinical development in advanced solid tumours; rationale mainly mitotic/taxane sensitisation.	Not DDR-selected.
CDK7—samuraciclib/CT7001	NCT03363893	Advanced malignancies, TNBC, castration-resistant prostate cancer and HR+/HER2− breast cancer, including fulvestrant combination	Phase I data reported one partial response in dose escalation, CBR 20% in TNBC expansion and CBR 36% with fulvestrant in HR+/HER2− breast cancer after CDK4/6 inhibitor therapy.	Not DDR-selected; breast-cancer/transcriptional-dependency context.
CDK7—SY-5609	NCT04247126	Selected advanced solid tumours; HR+/HER2− breast cancer with fulvestrant; pancreatic ductal adenocarcinoma with gemcitabine ± nab-paclitaxel	Phase I/expansion clinical development; public evidence mainly early-stage/trial-in-progress or abstract-level.	Not DDR-selected.
CDK9—alvocidib	NCT02520011 and related AML studies	Relapsed/refractory AML with MCL-1 dependence assessed by mitochondrial/BH3 profiling	A prospective biomarker-based AML study showed feasibility of MCL-1-dependence-based stratification and reported CRc rates in MCL-1-dependent relapsed/refractory AML.	Biomarker-driven, but not DDR-selected; biomarker is apoptotic dependence rather than DDR/HRD.
CDK9—AZD4573	NCT03263637; NCT04630756	Relapsed/refractory haematological malignancies, including combination studies with other anti-cancer agents	Phase I/combination clinical development in haematological malignancies; rationale centres on CDK9-mediated MCL-1 depletion.	Not DDR-selected.
GSK3β—LY2090314	NCT01287520; NCT01632306; NCT01214603	Advanced/metastatic cancer, metastatic pancreatic cancer combinations and acute leukaemia studies	Early clinical oncology development; pancreatic cancer study records and leukaemia studies do not establish DDR-selected efficacy.	No DDR-/HRD-selected clinical validation.
SFKs—dasatinib; bosutinib	Approved clinical use in Ph+ CML and related haematological indications	BCR–ABL/Src-family kinase inhibitor use in haematological malignancies	Approved status supports druggability and clinical feasibility of SFK/BCR–ABL targeting, but not DDR-context efficacy.	No DDR-selected validation; DDR relevance remains mainly extrapolated from signalling and preclinical sensitisation/resistance studies.

Note: This table is intended to clarify the clinical context of selected agents listed as approved or clinical stage in Table 3. The listed trials are representative rather than exhaustive. In most cases, clinical studies were conducted in tumour-type-defined or pathway-defined populations rather than in DDR-defective, HRD-selected or stress-phenotype-selected cohorts; therefore, clinical-stage development should not be interpreted as evidence of DDR-context clinical validation. All abbreviations used in the table are defined in the Abbreviations section.
